# Speed dependent phase shifts and gait changes in cockroaches running on substrates of different slipperiness

**DOI:** 10.1186/s12983-017-0232-y

**Published:** 2017-12-06

**Authors:** Tom Weihmann, Pierre-Guillaume Brun, Emily Pycroft

**Affiliations:** 1 Department of Animal Physiology, Institute of Zoology, University of Cologne, Zülpicher Strasse 47b, 50674 Cologne, Germany; 2 Ecole Normale Supérieure de Lyon Département de Biologie, Lyon, France; 3 Department of Zoology, University of Cambridge, Downing Street, Cambridge, CB2 3EJ UK

**Keywords:** Leg coordination, Body dynamics, Biomechanics, Poly-pedal locomotion, Insect, Arthropod

## Abstract

**Background:**

Many legged animals change gaits when increasing speed. In insects, only one gait change has been documented so far, from slow walking to fast running, which is characterised by an alternating tripod. Studies on some fast-running insects suggested a further gait change at higher running speeds. Apart from speed, insect gaits and leg co-ordination have been shown to be influenced by substrate properties, but the detailed effects of speed and substrate on gait changes are still unclear. Here we investigate high-speed locomotion and gait changes of the cockroach *Nauphoeta cinerea,* on two substrates of different slipperiness.

**Results:**

Analyses of leg co-ordination and body oscillations for straight and steady escape runs revealed that at high speeds, blaberid cockroaches changed from an alternating tripod to a rather metachronal gait, which to our knowledge, has not been described before for terrestrial arthropods. Despite low duty factors, this new gait is characterised by low vertical amplitudes of the centre of mass (COM), low vertical accelerations and presumably reduced total vertical peak forces. However, lateral amplitudes and accelerations were higher in the faster gait with reduced leg synchronisation than in the tripod gait with distinct leg synchronisation.

**Conclusions:**

Temporally distributed leg force application as resulting from metachronal leg coordination at high running speeds may be particularly useful in animals with limited capabilities for elastic energy storage within the legs, as energy efficiency can be increased without the need for elasticity in the legs. It may also facilitate locomotion on slippery surfaces, which usually reduce leg force transmission to the ground. Moreover, increased temporal overlap of the stance phases of the legs likely improves locomotion control, which might result in a higher dynamic stability.

**Electronic supplementary material:**

The online version of this article (10.1186/s12983-017-0232-y) contains supplementary material, which is available to authorized users.

## Background

Alternating sets of synchronously active diagonally adjacent legs, i.e. tripods in insects and four-leg sets in spiders, are largely regarded as the dominating coordination pattern employed by fast running insects and arachnids [1–3]. The sets either comprise the legs L1, R2, L3 (and R4) or R1, L2, R3 (and L4) with L and R indicating the left and right side legs, respectively, both counted fore to aft. These sets are accounted as the physical basis for spring-mass like dynamics as observed in the locomotion of some species [4–6]. Strictly alternating sets of legs are characterised by anti-cyclic activity of adjacent ipsilateral legs and the contralateral legs of the pairs of legs, i.e. by phase shifts of 0.5 [7–9]. Deviations from the alternating pattern [10–14], however, have rarely been analysed in the context of physical and biomechanical constraints and were mostly attributed to anatomical differences or inherent variability of poly-pedal locomotor systems. Nevertheless, characteristic changes in the speed-dependent increase of stride frequencies and oxygen consumption with simultaneously lacking aerial phases, as found in some studies [2, 15–17], seem to indicate an additional gait transition for high running speeds in insects and spiders.

In bouncing gaits such as running, trotting and multi-legged equivalents, significant proportions of movement energy can be elastically stored in the initial and recovered in the final part of the stance phase with optimised leg properties [18], which helps to economise locomotion. However, the stiffness of energy-storing elastic components such as sclerites, apodemes or other skeletal structures [19–21] must be matched to each other in all legs involved to provide concerted loading and unloading rates. In cockroaches, hind legs are characterised by distal joints with axes in parallel with the main ground force direction, which facilitates passive elastic energy storage in the hip joint [20].

Bouncing gaits, like trot and, with some restrictions, also gallop, are characterised by rhythmic upwards and downwards movements of the COM with in-phase oscillations of kinetic and potential energy [22, 23]. In running and trotting, the initial downwards movement is reversed by a single impulse of one leg or a set of synchronously active legs [4]. In gallop-like footfall patterns of vertebrates, such concerted stance phases decompose in consecutive ground contacts of the single legs [23], which affects locomotion dynamics and energy efficiency.

The majority of insect species seem to have significantly lower maximum running speeds than specialist runners, which are in the focus of many existing studies [24–28]. One of these specialists is the blattid cockroach *Periplaneta americana* which is characterised by extraordinarily long legs and a linear increase of the stride frequency over a wide range of running speeds [29]. Non-specialist runners, such as the blaberid cockroach species *Blaberus discoidalis* and *Nauphoeta cinerea* seem to be limited in their maximum leg cycle frequency but must still be able to attain high running speeds since successful predator avoidance is also crucial in these species. Just like in bipeds [30] and quadrupeds [31–33], the dependencies of stride frequencies to running speed are curvilinear in these blaberid cockroach species (cp. [2, 15]), which seems to indicate a gait change also occurs for these insects. However, the physical basis for the suspected gait transition has not yet been revealed. In a recent study on arachnid locomotion [34] we were able to show that fast moving mites employ temporally distributed footfall patterns at maximum running speeds and that such patterns may increase locomotion efficiency in arthropods. In nature, insects are commonly faced with slippery or unsteady substrates. Such surfaces do not provide secure footholds and can lead to unpredictable perturbations [35–37]. To our knowledge, no studies considering coordinative adaptations to slippery and unsteady substrates exist for fast moving arthropods. Accordingly, we examine here whether or not the saturating stride frequencies found in blaberid cockroaches are accompanied by changed footfall patterns and how they can affect running efficiency and endurance at high speeds on substrates with different grit sizes and slipperiness.

## Methods

### Animals

Thirteen adult male *Nauphoeta cinerea* (body length: 27 ± 1.3 mm, mean ± s.d.) were taken from a laboratory colony. This species has been examined with regard to their capabilities in substrate attachment and climbing in a couple of previous studies (e.g. [38]) which makes them a good model to examine the impact of substrate properties on locomotion. Moreover, *N. cinerea* is closely related to *Blaberus discoidalis*, whose locomotion has been extensively examined in the past (e.g. [20, 39–41]). Accordingly, our results can be well compared to those of the anatomically and behaviourally similar species.

**Fig. 1:**
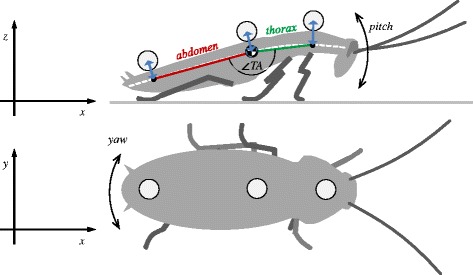
Sketch of a running cockroach drawn after a single frame of a typical sequence. Upper row: Side view of a specimen with markers on the pronotum, the metathorax and the caudal tip of the abdomen. The lateral edge along the body (white dashed line) was used to define the centre-line onto which the positions of the markers were projected (blue arrows). The projection of the thoracic marker was assumed as the position of the centre of mass. The position of the thorax was defined by the connecting line between the projections of the pronotum and the thorax markers (green line) while the position of the abdomen was defined by the connecting line between the projections of the thorax marker and the abdomen marker onto the mid-line (red line). The angle between thorax and abdomen was ∠ *TA*. For clarity only the legs of one tripod (R1, L2, R3) are shown. Lower row: Top view of a specimen with markers applied to the pronotum, the thorax and the abdomen. Both sets of legs are in contact with the ground, with the tripod made up by R1, L2 and R3 pictured just before taking off and the tripod consisting of L1, R2 and L3 having just touched down

The insects were kept in plastic containers at 25 °C and were supplied with dog food and water ad libitum. We used males only to prevent potential bias due to gravid females. When selecting study animals, we excluded cockroaches that were unwilling to run. All wings (representing about 2% of the body mass) were removed and three hollow Styrofoam spheres (2.5 mm diameter), coated in white paint were glued (using a mixture of paraffin and pine resin) onto the pronotum, the second to third thoracic tergum and close to the end of the abdomen, to provide markers for digitization (Fig. 1). The animals were allowed to recover for at least one hour before the trials. The total mass of the three spheres and the wax resin mixture amounted to 6.5 ± 1.2% of the wingless animals’ body mass. With markers, the mass of the animals was 450 ± 76 mg.

### Running track

Cockroaches were tested in a running track of 50 cm length and 3.5 cm width, with a floor covered with sandpaper of 30 μm particle size (Ultra Tec, Santa Ana, CA, USA). The walls were 5 cm high and covered with slippery 1 μm particle size sandpaper to prevent the animals from escaping. The central hardboard section (12 cm long) of the walkway could be exchanged for another piece. The pieces were either covered with non-slippery 30 μm particle size sandpaper or semi-slippery 12 μm particle size sandpaper. Different asperity sizes can facilitate or interfere with the abilities of claws or tarsal attachment pads to engage with a substrate. Micro-rough substrates within a range from some tenths to a few μm particle size impedes both, the claws and the attachment pads, from achieving good traction, which results in reduced friction and a reduced ability to climb [42–44]. Whether or not a substrate was slippery for *N. cinerea* cockroaches was tested on vertical substrates of 0.1, 1, 3, 12 and 16 μm particle size. While the specimens could not climb on 0.1 to 3 μm sandpaper and had difficulty walking on 12 μm sandpaper, they coped with 16 μm sandpaper. In the central region, the walls of the running track were made of Perspex allowing video recording of the kinematics in the lateral view (see below). All examined runs were escape runs. The insects were spurred by short puffs of air or by touching their feet or cerci with a fine paint brush. Recordings of straight and continuous runs were saved for further analysis.

### Recording and digitisation

The runs were recorded in top view with a Photron FastCam SA2 (Photron Ltd., Tokyo, Japan, resolution 2048 × 2048 pixels) high-speed video camera which was oriented perpendicularly to the walkway. In the central region of the running track with the exchangeable surface and the Perspex walls, a bracket with a 45° mirror provided a side view of the running insects.

Recordings were made at 250 frames per second and with a shutter time of 2 ms. The central region of the track filled the camera’s field of view, resulting in a spatial resolution of approximately 17 pixels per mm. The track was illuminated with two white LED lights (XS40, Spectrum Illumniation Inc., Montague MI, USA) fitted with red filters to reduce their brightness in the spectral range visible to the insects.

**Fig. 2:**
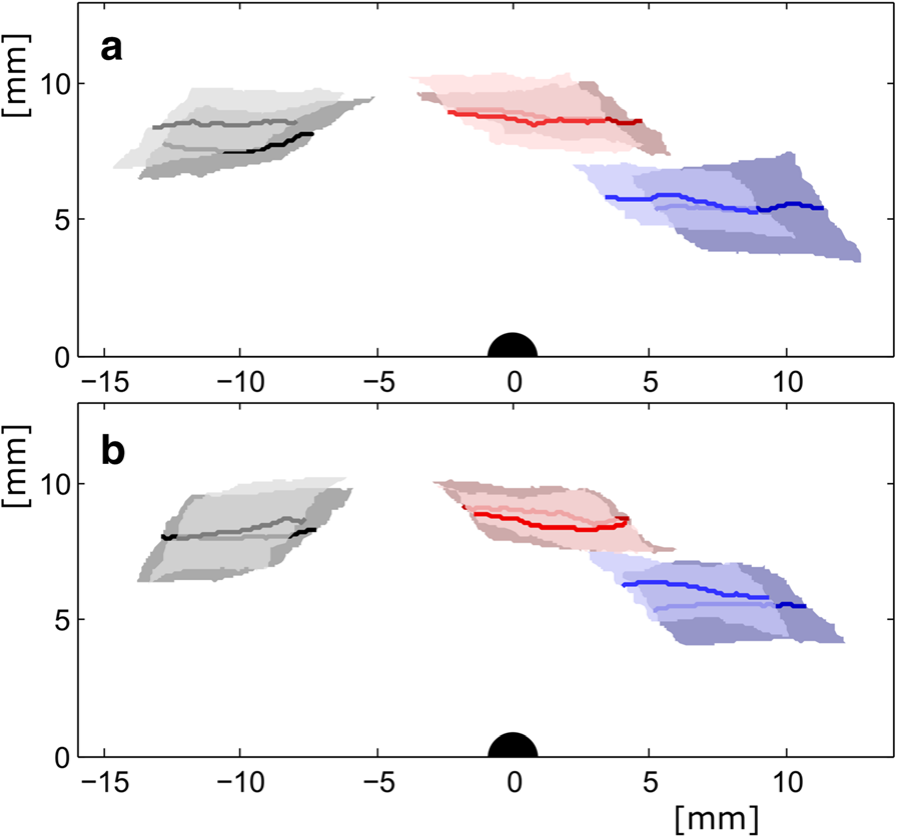
Contact areas for metachronal (dark sublayer) and alternating (bright upper layer) gait patterns on non-slippery and slippery substrate. **a** Averaged contact positions on the non-slippery substrate with median trajectories of the legs’ tarsi (dark lines in centre of each layer) with respect to the COM (black semi-circle at the point of origin). Fore legs are depicted in blue, middle legs in red and rear legs in black. Contact areas are bound in anterior-posterior direction by the medians of the anterior and posterior extreme positions for a given lateral distance to the COM. In the lateral direction, the boundaries are the 25% and the 75% quartiles of the lateral distributions for distinct anterior-posterior positions of the tarsi (see [16]). **b** Averaged contact areas on slippery substrate with median trajectories of the legs’ tarsi with respect to the COM

The videos were digitised in ProAnalyst Lite Edition (Xcitex Inc., Massachusetts, USA). A 72 × 16 × 9.6 mm Lego™ brick assembly was used for calibration. Top and side views were calibrated separately taking into regard slight aspect variations caused by the mirror. Coordinate axes were adjusted such that the fore-aft axis was aligned to the direction of the walkway. The three marker points on the pronotum, thorax, and abdomen were tracked automatically. Additionally, in the top view, manual tracking was used to digitise the positions of the foot tips (Fig. 2) during contact (cp. [16]). Only straight runs with constant running speed and where animals did not touch the walls were considered. Runs in which the running speed varied by more than 10% were excluded from analyses.

### Analyses

**Fig. 3:**
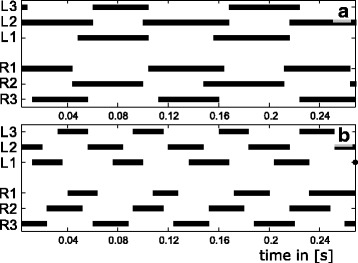
Example gait patterns; bars depict ground contacts of the legs which are counted fore to aft with L indicating left and R indicating right side legs. **a** Alternating tripodal run at an average speed of 0.1 ms^−1^ with an average phase shift between ipsilateral fore and rear legs of 0.91 and an average duty factor of 0.52. **b** High speed metachronal run at an average running speed of 0.24 ms^−1^ with an average phase shift between ipsilateral rear and fore legs of 0.71 and an average duty factor of 0.41

**Fig. 4:**
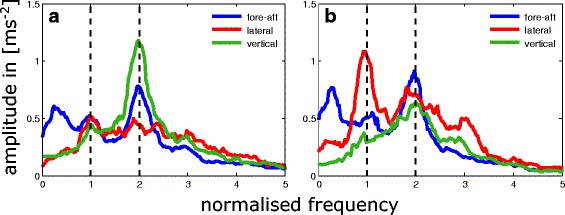
Median trends for the frequency spectra of COM accelerations in fore-aft (blue), lateral (red) and vertical (green) direction in ms^−2^. All frequencies were normalised by dividing specific values by the corresponding stride frequency. **a** Runs with alternating tripodal leg coordination **b** Runs with metachronal leg coordination. Substrate slipperiness did not affect the graphs and runs of both conditions were merged

In order to assess the relative frequencies of the different leg coordination schemes; initially all recorded sequences were roughly classified. To this end, it was determined whether the ground contacts of front and rear legs of one side were in phase; in this case they were classified as alternating tripod gait (Fig. 3a). Traditionally metachronal leg coordination, has been defined by reference to slow movement sequences or those with high duty factors (e.g. [9, 45, 46]) at which the wave travels down the entire grouping of appendages. Wave propagation appears somewhat less clear when duty factors decrease and relative fluctuations in touch down and take off increase. However, for bouncing gaits, such as trotting and the multi-legged equivalents, synchronised sets of legs, such as tripods, represent the functional unit which becomes inoperative when the contact phases of the single legs dissipate. As a direct consequence, such a temporal dissipation results in a loss of the typical vertical body oscillations (see Fig. 4). Accordingly, in order to simplify the terminology of the new high speed gait and because of the considerable similarity of the gait patterns (Fig. 3b) all runs with low synchronicity between front and rear legs of one side were classified as metachronal.

Ninety runs were recorded on the non-slippery and 81 runs on the slippery substrate. 11 runs were classified as metachronal (12%) on the non-slippery substrate, and eight on the slippery substrate (10%). Metachronal runs seemed to occur predominantly at high running speeds.

In order to increase the sample size of metachronal runs allowing statistical comparisons between the paradigms, 20 additional fast runs were recorded. Finally, 24 of the best runs, spanning the full speed range of the examined runs, were digitized and analysed for the non-slippery condition; 11 of them being alternating tripodal gaits and 13 metachronal. On the slippery substrate 25 runs were analysed with 12 being alternating and 13 metachronal. The ratios between metachronal and tripodal runs implicitly indicate that not all animals could be urged to run quickly. Eventually, the samples cover 6 to 8 individuals which contributed one or two high quality runs. For each run a sequence of 3.6 ± 1 consecutive strides was digitised.

The phase shift between the touch-downs of the ipsilateral front and rear leg was used to quantitatively distinguish alternating tripod gait from metachronal gait patterns. Runs were classified as metachronal if the median phase values of a run differed by more than ±0.2 from synchrony (indicated by a phase value of 0 or 1) (cp. [8]). Differences between phase relations were tested for significance using the Kuiper two-sample test which is a circular analogue of the Kolmogorov-Smirnov test [47].

Before analysis, a correction was applied to the side view data to account for differences in the heights of the markers (within and between specimens) due to variation in the amount of wax used to apply each marker. The dorsal cuticle of *N. cinerea* narrows towards a visible lateral edge (Fig. 1a). This anatomical structure was easily visible in the lateral view and was taken to be the dorsal-ventral centre line. The distances between the centres of the markers and this midline were determined for each individual. Then, the centre points of the markers were projected orthogonally onto the centre line. Since the COM of the animals was on an anterior-posterior level with the hind limb coxae, the thoracic marker was just in front and very close to the COM of the cockroach body. Therefore it was taken as its representative.

Once the position of the COM was obtained in the side view further parameters could be extracted in a right handed system of coordinates. Extracted parameters included the height of the COM above the substrate, distance covered per stride, pitch, yaw and the angle between the thorax and abdomen. Fore-aft, lateral and vertical velocities as well as accelerations of the body were then calculated using the derivative of the COM position data and by smoothing the primary data by fitting gliding second-order polynomials to the time series, including four adjacent points on both sides at each position [48]. Pitch was calculated in the side view as the angle between substrate and the connecting line between the pronotum marker and the thorax marker. To calculate yaw, the line connecting the pronotum marker and the abdominal marker in the top view was used. The connecting lines between pronotum and thorax marker and between thorax and abdomen marker were used to calculate the ventral angle between thorax and abdomen in side view. All body oscillations were referred to the stride periods of the second legs; lateral data was inverted for tripods consisting of L1, R2 and L3 such that all data was normalised onto the reverse set of legs (R1, L2, R3).

Moreover, kinematic parameters of the legs such as stride frequency (*f*_*T*_), duty factors (*β*), contact and swing rates (*t*_*C*_^*−1*^ and *t*_*S*_^*−1*^) and the positions of the legs in contact with the ground (see [16]) were also examined. Touch-down and take-off positions as well as the lateral distance of the feet to the COM were extracted for all legs while the second legs were used as proxy for the analyses of the distances travelled by the COM during contact with the ground (*s*_*C*_) and during a whole stride (*s*_*T*_). Fore-aft and lateral distances of the feet with respect to the COM (Fig. 2) were measured along the x- and y-axis of a body-fixed coordinate system in top view. Data for corresponding left and right legs were merged.

**Fig. 5:**
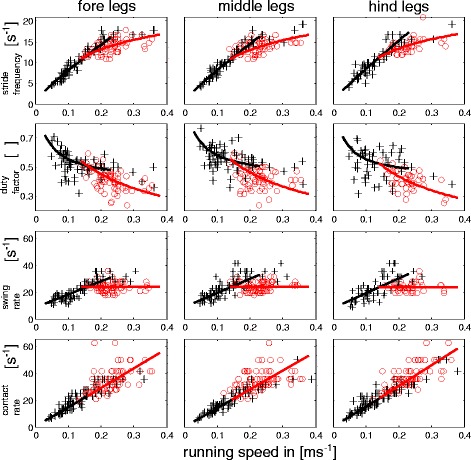
Stride frequencies, duty factors, swing rates and contact rates plotted against running speed for all walking legs and non-slippery conditions. First row: Stride frequency (*f*_*T*_); Second row: duty factors (*β*); Third row: swing rates (*t*_*S*_^*−1*^); Fourth row: contact rates (*t*_*C*_^*−1*^). Red circles are measured values for runs with metachronal leg coordination while black crosses depict values from alternating tripodal runs. Black and red lines are linear least squares regressions in the third and fourth rows while these regressions were used to calculate the approximation lines in the first two rows (further explanations can be found in the methods section). Red approximation curves refer to metachronal and black to alternating runs

The changes in contact duration (*t*_*C*_) and swing duration (*t*_*S*_) with speed are highly non-linear (Additional file 1: Figure S8). Both measures can be linearized by considering the reciprocal values (*t*_*C*_^*−1*^ and *t*_*S*_^*−1*^), i.e. contact and swing rate. The dependencies on running speed were then approximated by using linear least squares fits. The contact rate increases linearly with running speed. On the non-slippery substrate the swing rates have sloped dependencies at lower speeds and adopt constant values at high speeds. The point of intersection indicates a transitional velocity (Fig. 5).

Stride frequencies and duty factors derive from these durations and rates. Their velocity dependencies are non-linear as well. Their approximate speed dependencies were calculated by following Weihmann [16]. Thus, stride frequencies (*f*_*T*_) were calculated as *f*_*T*_ = *t*_*T*_^*−1*^ = (*t*_*C*_ + *t*_*S*_)^−1^. Their slopes are linear and relatively steep at low speeds and decrease in a non-linear manner at speeds above the transition point (Fig. 5). With *t*_*T*_ being the stride duration, i.e. the reciprocal of the stride frequency, duty factors were calculated conventionally (*β* = *t*_*C*_/*t*_*T*_) for all pairs of legs. Due to the specific characteristics of *t*_*C*_ and *t*_*T*_, their velocity dependencies displayed graphs consisting of two adjacent hyperbola for each leg. The curvatures of the graphs were higher at low running speeds and low above the transition speed (Fig. 5).

On the slippery substrate, metachronal runs occurred at much lower running speeds which resulted in a wide overlap region (Additional file 1: Figure S6). Therefore, no saturation of stride frequencies and swing rates could be observed.

All analyses were performed using MatLab scripts (MATLAB 7.10.0; The MathWorks, Natick, MA, USA). All values are provided either as mean ± s.d. or as median and the 25% and 75% percentiles (Q25/Q75). Accordingly for pairwise comparisons, t-tests or non-parametric tests such as the Mann-Whitney U test were used. For multivariate analyses one way analyses of variance (ANOVA) with Tukey-Kramer post-hoc tests were applied. If not specified otherwise, all statistical tests refer to a 5% significance level.

Oscillations of the COM accelerations were analysed in all three dimensions using Matlab’s Fast Fourier Transformation (FFT) algorithm. Overall ground reaction forces of the walking legs determine the accelerations of the animal’s body. Therefore, body oscillations provide insight into the application of leg forces over time. The frequency spectrums of the acceleration amplitudes were calculated for each running sequence and frequency values were normalised due to division by the individual stride frequency of each run (Fig. 4).

**Fig. 6:**
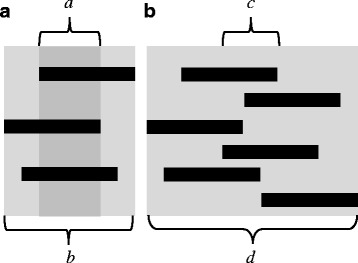
Sketches explaining the calculation of the tripod synchrony factor (TSF) and the temporal overlap ratio between consecutive sets of legs. **a** The TSF is calculated as: *TSF* = *a*/*b*. **b** The temporal overlap between consecutive sets of legs is calculated as *c*/*d*. Black bars depict intervals with legs in contact with the ground

In order to assess the functional basis of the COM oscillations, tripod synchrony factors (TSF) were determined in accordance with the method proposed by Spagna et al. [49], i.e. as a normalised fraction of contact phase overlap between legs in the same set of legs (Fig. 6a). Consequently, the synchrony factor adopts values between 0 and 1, at which 1 corresponds to perfect synchronicity. Additionally, we determined the relative temporal overlap of consecutive stance phases of the sets of legs. In contrast to the “Per cent double support phase per stride” introduced by Ting et al. [2] we did not focus on those phases with six legs on the ground but determined the time span of a set’s ground contact from the first contact of one of the legs till the last leg of the set has lost ground contact. The fraction with at least one leg of both sets in contact with the ground, was then related to the overall time span of the ground contacts of the two sets of legs (Fig. 6b).

By using the 3D fluctuations of the COM over the strides of the second legs and the average mass, the specimens’ fore-aft (*E*_*kx*_), lateral (*E*_*ky*_) and vertical (*E*_*kz*_) kinetic energies as well as the potential energy (*E*_*p*_) of the COM were calculated. The kinetic energies were calculated as the product *0.5·m·v* ^*2*^ with *m* being body mass and *v* being fore-aft, lateral or vertical velocities respectively. The gravitational potential energy was calculated as the product *m·g·h* where *m* is the body mass, *g* represents acceleration due to gravity and *h* is the vertical distance between COM and the substrate. According to Full and Tu [25], the mass specific external mechanical energy per unit distance (*M*_*COM*_) was then calculated by relating the sum of the changes in these COM energies to the distance travelled during a stride, which is equivalent to the external mechanical work and has units of J·kg^−1^·m^−1^ [25, 31, 50].

$$ {M}_{COM}=\frac{\Delta  {E}_{kx}+\Delta  {E}_{ky}+\Delta  {E}_{kz}+\Delta  {E}_p}{body mass\bullet stride length} $$

In order to assess locomotion dynamics, recovery and congruity for *E*_*kx*_ and *E*_*p*_ were calculated for the stride periods of the second legs. Recovery provides information about the percentage of exchange between kinetic and potential energy and was calculated in accordance with Ahn et al. [51]. Thus, high percentages would indicate out-of-phase fluctuations of the two energy forms, i.e. COM dynamics similar to walking. Low percentages would indicate in-phase fluctuations as similar to running [31]. Congruity, in turn, compares the curve progression of kinetic and potential energy. Values of 100% occur if both trajectories follow the same trend throughout a whole gait cycle and would indicate a running gait; opposed curve progressions would result in low values, indicating walking gaits [51, 52].

## Results

### Running speed

Maximum running speed was reduced on the slippery substrate. While values of up to 0.35 ms^−1^ were observed on non-slippery the animals reached only 0.25 ms^−1^ on the slippery substrate (Table 1 in Appendix). Maximal attainable stride frequencies of about 15 s^−1^ did not differ between slippery and non-slippery substrates (Fig. 5, Additional file 1: Figure S6). The values are very similar to those observed in *Blaberus discoidalis* (cp. Fig. 7 in [15]).

On coarse substrate, running speed was significantly higher in metachronal runs (mean: 0.21 ms^−1^; Q25: 0.17 ms^−1^/Q75: 0.24 ms^−1^), while alternating tripodal patterns were used predominantly at lower running speeds (0.12 ms^−1^; 0.09/0.2 ms^−1^) (cp. Table 1 in Appendix). Running speeds of metachronal runs on non-slippery substrate were also significantly higher than those of the slippery conditions while the other groups did not differ from each other.

### Posture

In the working range of the fore legs, significant shifts in anterior direction occurred during metachronal coordination with respect to alternating tripodal runs (Fig. 2; Additional file 1: Table S1). The working ranges shifted for about 1.3 mm on slippery and 2.4 mm on non-slippery substrate. Positional shifts in the second and third legs were smaller and barely significant. Contact lengths (*s*_*C*_) were significantly smaller in alternating tripodal runs on slippery substrate. In the other conditions no differences could be observed.

The average height of the COM did not change significantly with substrate properties or leg coordination; it was always about 3.74 ± 0.48 mm (Table 1 in Appendix).

### Temporal measures

On the non-slippery substrate, contact rates (*t*_*C*_^*−1*^) increased linearly with speed (Fig. 5); the slopes (145/140/153) were similar for front, middle and rear legs and no change could be observed between alternating and metachronal leg coordination. Swing rates (*t*_*S*_^*−1*^) also had similar slopes (98/104/113) and y-intercepts (8.4/9/7.7) at lower speeds, i.e. with alternating tripodal leg coordination. At high running speeds and metachronal leg coordination, swing rates were constant and very similar among the legs (24 ± 3.5 s^−1^/ 24.3 ± 3.7 s^−1^/ 23.7 ± 3.4 s^−1^).

### Phase relations of the legs

As expected, on non-slippery substrate, *N. cinerea* showed ipsilateral phase shifts of about 0.5, i.e. alternating sets of legs at lower and intermediate running speeds. The circular mean of the phase lag of front leg touch-downs in the stride period of middle legs (_1_L_2_ following the nomenclature of Shultz 1987 [53]) was 0.49 with a confidence interval (CI) between 0.47 and 0.51. The value for the middle legs with regard to rear legs (_2_L_3_) was 0.45 (CI: 0.42/0.47) and the contralateral phases of the rear legs were about 0.5 (0.48/0.51) (Additional file 1: Figure S7, Additional files 2 and 3). At high running speeds, however, ipsilateral phase shifts between the front and middle legs were about (0.32; 0.29/0.34) and deviated significantly (*p* < 0.001) from 0.5. Accordingly, front and rear legs did not act synchronously and the legs of each set made ground contact successively rather than synchronously (Fig. 3b). Thus, coordination patterns were metachronal rather than alternating at high running speeds, though contralateral phases of the rear legs were still about 0.5 (0.48/0.51). On slippery substrate phase values for alternating (_1_L_2_: 0.48; 0.46/0.5, _2_L_3_: 0.42; 0.41/0.44) and metachronal (_1_L_2_: 0.33; 0.31/0.36, _2_L_3_: 0.4; 0.38/0.42) runs were similar to those on non-slippery substrate (Additional file 1: Figure S7).

**Fig. 7:**
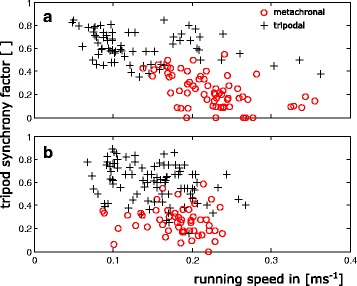
Tripod synchrony factors (TSF) after Spagna et al. [49] plotted against running speed. Red circles refer to runs with metachronal leg coordination and black crosses to tripodal runs. **a** Non-slippery conditions **b** Slippery conditions, which led to lower maximum speeds and strong overlap in the speed ranges of tripodal and metachronal runs

In accordance with the changed phase lag between front and middle legs the tripodal synchrony factor (TSF) also differed between alternating tripodal and metachronal runs. While the TSF adopted values of about 0.61 ± 0.15 (*n* = 161) in alternating runs, the values decreased when the animals used metachronal leg coordination (0.25 ± 0.13; *n* = 119; see Fig. 7), i.e. at higher running speeds (*p* < 0.001).

### Duty factors and temporal overlap of leg activity

The speed dependencies of the duty factors were similar in all pairs of legs. Duty factors decreased from about 0.7 at low running speeds to values around and below 0.5 at maximum running speeds (Figs. 3 and 5).

Although duty factors reached relatively low values at high running speeds on non-slippery substrate and even fell below the critical value of 0.5, the consecutive footfalls of the legs of a set and increased temporal overlap of the consecutive sets of legs, prevented periods without contact with the ground. For runs above 0.15 ms^−1^ running speed, the temporal overlap of the consecutive sets of legs was significantly higher (*p* < 0.001) in runs with metachronal leg coordination (0.31 ± 0.11; *n* = 106) compared to alternating tripodal runs (0.23 ± 0.1; *n* = 72). Below the transitional speed of 0.15 ms^−1^ metachronal leg coordination was effectively lacking; in alternating runs the temporal overlap adopted values of 0.29 ± 0.12 (*n* = 87) which did not differ from the values for metachronal runs at higher running speeds.

### Body oscillations

The FFT analyses of the COM accelerations revealed differences between alternating and metachronal runs in vertical and lateral direction, while the frequency spectrum in fore-aft direction differed only little (Fig. 4). On slippery and non-slippery substrate, tripodal runs were characterised by relatively high vertical and low horizontal amplitudes while metachronal runs had lower vertical and higher lateral amplitudes. In tripodal and metachronal runs, the frequency-maxima of the fore-aft and vertical oscillations corresponded to two times the stride frequency while the maxima of the lateral oscillations were equal to stride frequency.

In all examined sequences, recovery was low (0.09; 0.03/0.16) and congruity was always quite high (0.7; 0.59/0.81). Running speed and leg coordination did not have any impact.

On both substrates, the external *M*_*COM*_ was significantly higher in tripodal than in metachronal runs (1.77 ± 0.78 J· kg^−1^·m^−1^ vs. 1.47 ± 0.69 J· kg^−1^·m^−1^; Table 1 in Appendix). The differences were about 20% on non-slippery and 10% on slippery substrate. This coincides with differences in the peak-to-peak amplitudes of the vertical oscillations, which were also significantly decreased in metachronal runs (0.51–0.63 mm) compared with tripodal runs (0.94–1.02 mm) (Table 1 in Appendix). The lateral amplitudes were not different on the 5% significance level. However, on slippery substrate, with metachronal leg coordination, lateral peak-to-peak amplitudes (1.42 mm) tended to be smaller than for the other conditions (1.55–1.73 mm; *p* = 0.0506; Table 1 in Appendix).

On average the COM changed its position relative to the running direction once during a stride in metachronal and tripodal runs (Additional file 1: Figures S1-S4). Though lateral oscillation patterns were not particularly consistent, during alternating runs, the position of the COM swayed predominantly to the side of the body with the front and rear legs on the ground while the COM swayed to that side with the second leg in contact with the ground during metachronal runs. Only in metachronal runs on slippery substrate were we able to find distinct lateral oscillation patterns. Concurrently, the average trajectory of the yaw angle was markedly sine shaped here (cp. [54]) while it was rather cosine shaped during alternating runs on non-slippery substrate. Accordingly, at touch down, yaw angles started with low values in metachronal runs on slippery substrate while in alternating runs on non-slippery substrate yaw angles were maximal when the legs touched the ground. The trajectories of the yaw angle seemed generally rather sine shaped on slippery and rather cosine shaped on non-slippery substrate (Additional file 1: Figure S5).

The mean pitch and yaw angles (Fig. 1; Additional file 1: Figure S5) were about zero in all running conditions. However, the pitch amplitudes were significantly lower in metachronal runs (about 4.5°) compared to alternating tripodal runs (6° to 7.5°). This difference was higher on non-slippery substrate (Table 1 in Appendix). The amplitudes of yaw were not significantly different from each other and ranged from 6.8° to 8.1° (Table 1 in Appendix). The mean angles between thorax and abdomen were between 160° and 173° and significantly higher in alternating tripodal runs than in metachronal runs (Table 1 in Appendix). Accordingly, the flexion of the cockroaches’ body was more pronounced in metachronal runs and their position was relatively outstretched in tripodal runs. The amplitudes of this angle (4.1° - 4.7°) were not different between the running conditions.

## Discussion

**Fig. 8:**
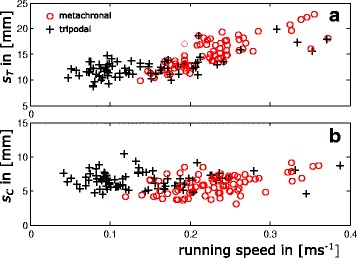
Distances covered by the COM during stride and contact duration of the second legs. **a** Stride length (*s*_*T*_) of the second legs. Red circles refer to runs with metachronal leg coordination while black crosses refer to alternating tripodal runs **b** Distances covered by the COM during leg contact (*s*_*C*_)

In ambulatory locomotor systems, gaits and gait changes have been widely examined in two- and four-legged vertebrates. Arthropods, in turn, are still largely regarded as being restricted to only two different gaits; namely a highly feedback-controlled slow walking gait with metachronal leg coordination and the rather feedforward controlled running gait which is characterised by alternating sets of diagonally adjacent legs [10, 15, 39, 55]. Indeed, some species such as wood ants or fruit flies seem to use only these gaits [11, 52]. In these species the tripodal synchrony factors (TSF) are low at very low, increase at intermediate and reach a plateau of high values at high running speeds. However, our results clearly show that *N. cinerea* exhibits two fast running gaits which supplement the typical slow and mostly unsteady metachronal gait. Thus, in *N. cinerea* the TSF values decrease again at running speeds higher than 0.15 ms^−1^ (Fig. 7). Reduced synchronicity, in turn, is caused by phase shifts between the ipsilateral legs that deviate significantly from 0.5. Since phase shifts are relatively constant for each co-ordination pattern, gradually decreasing TSF values are caused by speed-dependent decreasing contact durations (Fig. 5). The slopes of *t*_*C*_^*−1*^ against running speed and those of the contact lengths (*s*_*C*_; Fig. 8) are similar in all walking legs. Therefore, the different lengths of fore, middle and rear legs [41] apparently do not affect stride length and are not the reason for changed phase shifts at high running speeds.

Decreasing phase shifts and resulting consecutive ground contacts of the legs of a tripodal set facilitate permanent body support although swing durations increase relative to single leg contact durations, which enables further increasing stride lengths without the need for ballistic phases of the COM (Fig. 8). This is reflected by a significantly higher temporal overlap between consecutive sets of legs in metachronal runs if compared with tripodal gait patterns.

Constant swing durations limit the contraction speeds of the involved muscles and prevent excessive metabolic costs which would occur when the legs would be swung in decreasingly shorter intervals necessary for running with uniform, short strides and therefore largely increased stride numbers per distance covered [56]. Correspondingly, high speed metachronal leg coordination can limit the metabolic costs necessary for swinging the legs back to touch-down position and reduces the number of strides per distance travelled, which both contribute significantly to total running costs [57–59]. A reduction in stride numbers may be particularly significant in small legged organisms with their increased relative joint stiffnesses. When body size and limb weight decrease, relative cross-section areas of leg muscles and joint membranes increase. Accordingly, damping within these structures increases relative to inertial forces of the legs and several researchers hypothesized that movements of small animals are dominated by passive joint forces rather than inertia [60–62]. Even though, dissipation within the leg joints likely affects leg swing and accompanied metabolic costs, for cockroaches, our results indicate that stance phases seem to be governed by the inertia of the body which enables exploitation of the advantages of synchronized sets of legs such as high static stability and the employment of elastic energy storage at intermediate running speeds.

Since power is the product of force and contraction speed, muscles generate their highest power output at intermediate contraction speeds, which makes them particularly efficient in this range. Eccentric load, as typical for anti-gravity muscles during stance, is limited and quickly leads to overstrain [63, 64]. In spring-mass systems, vertical oscillation amplitudes and maximum vertical ground reaction forces increase with speed [65–67]. Our results in *N. cinerea* do also show pronounced vertical oscillations during alternating tripodal runs (Fig. 4, Additional file 1: Figures S1-S4, Table 1 in Appendix). Accordingly, at high running speed, the interplay of concordant body pitch, high vertical amplitudes and accompanied low reversal points may lead to unfavourable joint angles in the already bent fore and middle legs [41]. Moreover, excessive vertical amplitudes are prevented by the generally low position of the COM in cockroaches. High amplitudes and ground reaction forces would also lead to high contraction speeds in concentrically acting and high stresses in eccentrically loaded muscles. Therefore, further adherence to spring-mass dynamics at very high running speeds would be disadvantageous in particular as blaberid cockroaches use fast sprints only occasionally (cp. [39]), and seem rarely adapted to prolonged fast leg muscle activity.

Energy efficiency in bouncing gaits such as running, trotting and the alternating tripodal gait of insects such as cockroaches mostly relies on spring-mass dynamics. This requires matched stiffnesses of the energy storing components in all walking legs to enable concerted loading and unloading rates [34], particularly in poly-pedal locomotor systems. In blaberid cockroaches hind leg stiffness is largely determined by the passive mechanical properties of the legs themselves [20, 68]. Except for the coxa, the axes of all involved joints are almost in parallel with the ground force vectors, which makes cockroach hind legs particularly suitable for elastic storage and recovery of movement energy in vertical directions. As a result, the hip joint effectively stores and recycles displacement energy over a wide range of oscillation frequencies [20]. In the front and middle legs, however, the joint axes are perpendicular to the main ground force direction. Accordingly leg stiffness depends strongly on the activity of leg muscles and their contraction properties. Whether or not elastic mechanisms take effect in these legs has not been examined yet. However, to enable efficient use of the hind leg spring, stride frequencies and timing of the anterior legs have to be adjusted to that of the former.

COM accelerations represent causative overall ground forces. The FFT analysis of COM accelerations in *N. cinerea* revealed peaks at two-times stride frequency for vertical and fore-aft fluctuations and one-time stride frequency for lateral oscillations, which is in agreement with spring-mass running dynamics [16]. However, along with the changes in inter-leg coordination as discussed above the FFT analysis also revealed distinct spectral changes. Thus, in alternating tripodal runs the vertical amplitudes were significantly higher than in metachronal runs while lateral amplitudes were higher in the latter (Fig. 4). Accordingly, the major plane of COM oscillations changed from sagittal to horizontal.

Direct measurements also showed significantly higher vertical peak-to-peak amplitudes of the COM in alternating tripodal gaits (Table 1 in Appendix). Accordingly, the mass specific external mechanical energy was also significantly higher in alternating runs. However, bouncing gaits such as the alternating tripodal pattern of insects enable efficient loading of elastic leg structures and benefit from these structures’ elastic recoil. This internal energy storage and transmission system is not externally visible and increases the apparent energy fluctuations and total energetic costs while effective metabolic costs may be considerably lower [15, 40, 69].

In alternating runs the spectrum of lateral acceleration amplitudes was blurred and did not show a clear maximum (Fig. 4a). However, such a clear maximum close to one-time stride frequency is required by the model proposed by Schmitt and Holmes [70] to enable dynamic stability in the horizontal plane. Therefore, the dynamics assumed for their lumped two-legged model do better correspond to those observed for metachronal runs with their pronounced lateral oscillations and a clear spectral maximum. Since static stability is reduced with temporally distributed touch-downs within a set of legs, dynamically stabilizing effects imposed by COM dynamics similar to the lateral leg spring model [6] and distributed mechanical feedback [3] may replace static stability as the major stabilizing mechanism at high running speeds. This change towards passive stabilization and feed forward control can also be related to the reduced ratio between contact duration and the fastest possible reflex response [16, 71]. Thus, in the range of the transitional speed of about 0.15 ms^−1^ contact durations fall below 40 ms (Fig. 5) whereas the shortest reflex responses in cockroaches are about 20 ms [72]. Accordingly, half the stance duration has passed and the legs’ ground forces have reached their maximum before a reflex response could at all affect leg activity.

The mean angles between thorax and abdomen were slightly but significantly lower in metachronal runs (Table 1 in Appendix); the total difference was about 5°. Nevertheless, the positional change led to a smaller distance between the caudal abdomen tip and the substrate surface, which might result in intermittent ground contacts as found in wood ants [27] and may have a stabilizing effect for the vertical position of the COM. However, the bent body posture could also simply be the result of anatomical constraints in external leg muscles [73] that emerge at high running speeds.

### Comparative

*Blaberus discoidalis* is another blaberid cockroach species extensively used for experimental examinations. This species shows a similar saturation of the stride frequency with increasing running speed as *N. cinerea* while aerial phases are also lacking [2, 15]. In *N. cinerea*, stride frequency saturation is primarily caused by swing phases with constant and therefore relative to the contact phases increasing durations. Without significant aerial phases, constant swing phases, in turn, are indicative for changed leg coordination. Therefore, *B. discoidalis* seems to pass through the same gait change as *N. cinerea*.

For Namibian tenebrionid desert beetles Bartholomew et al. [17] reported constant oxygen consumption rates at running speeds above about 0.13 ms^−1^, while this rate increased linearly at running speeds below the transitional speed. Moreover, at high running speeds the beetles had very stable COM trajectories without visible height fluctuations [17]. This corresponds well with the reduced COM fluctuations found for *N. cinerea* when running with metachronal leg coordination, in particular on slippery substrate (see below). However, due to the flattened shape of the beetles’ body, lift, which would reduce vertical ground reaction forces, may also contribute to the constant cost of transport at high running speeds [17], while drag should play only a minor role at running speeds significantly below 1 ms^−1^ and given Reynolds numbers [24].

Apart from insects, gait changes from alternating to metachronal leg coordination also seem to occur in fast running arachnids. Thus, a change from linearly increasing to constant swing rates and a concurrently reduced increase of stride frequencies were also found in the vagrant spiders *Ancylometes bogotensis* and *Cupiennius salei* [16, 74]. Recent experiments in tiny mites with body lengths of only about 1 mm also revealed coordinative changes at their highest running speeds [34] which indicate dynamical changes even in some of the smallest terrestrial runners.

However, insects do not always change gaits when attaining high running speeds. The blattid cockroach *Periplaneta americana,* for example, is specialized in extremely fast escape runs. This species increases its stride frequency nearly linearly over a wide range of running speeds [29]. At their highest speeds they even lift the frontal legs off the ground and use only their hind legs for propulsion [25]. Accordingly, they cannot adopt metachronal leg coordination and seem to exploit other mechanisms to limit metabolic expenses. Interestingly, the mass specific external mechanical energy found for running *N. cinerea* (1.47 to 1.77 J·kg^−1^·m^−1^) is quite similar to values of about 1.5 J·kg^−1^·m^−1^ as measured for *P. americana* [25].

Linearly increasing stride frequencies were also found in wood ants and fruit flies [11, 52] while the fast running North African dessert ant *Cataglyphis fortis* reduces the increase of the stride frequency at very high running speeds [26]. In *C. fortis*, however, the synchronisation of the legs within a set is also maintained at high running speeds, and stride frequencies increase slower at high running speeds due to the occurrence of aerial phases.

According to [75] the jumping bristletail *Petrobius* reacts upon disturbances by jumps or a peculiar high speed jumping gait. Though no detailed data are available, this jumping gait indeed might reproduce mammalian gallop dynamics with three consecutively active symmetric pairs of legs instead of four sequenced legs making up a set which replaces itself after a ballistic phase and causes redirection of the COM from downwards to upwards. Leg coordination patterns similar to that of *Petrobius* were described recently for the relatively slow locomotion of some South African species of dung beetles [76]. The COM dynamics of these beetles’ “gallop” gait, however, rather seems to employ inverted-pendulum dynamics (e.g. [50, 77]) allowing them to travel at relatively low metabolic costs on deformable granular media [78].

### Impact of slipperiness

On slippery substrate the onset of metachronal leg coordination occurs at significantly lower running speeds (Table 1 in Appendix) and maximum running speeds were generally lower (Fig. 7) compared with non-slippery conditions. Accordingly, the slippery sand paper seems to prevent the transmission of high horizontal forces as necessary to propel the animals at velocities above 0.25 ms^−1^.

In insects using the alternating tripodal gait, the front and middle legs generate lateral ground reaction forces and brace against each other [52, 79]. In *N. cinerea*, desynchronization of the legs within the alternating sets as found in metachronal runs arises primarily from changing phase relations between these legs. Therefore, the early onset of metachronal coordination on slippery substrate may indicate active avoidance of lateral bracing of front and middle legs which might be of significant functional impact on coarse substrates. In principle, due to lateral bracing, elastic structures in the legs can be loaded that recoil in the second half of the contact phases and might contribute to energy recovery during a stride. Moreover, bracing can also be a mechanism to control the lateral dynamics of the COM and therefore dynamic stability (see [54]). However, on slippery and granular substrates lateral forces are difficult to transfer onto the ground which is also reflected by the significantly shorter contact lengths (*s*_*C*_) found in alternating runs on slippery substrate (Additional file 1: Table S1).

Along with a significant reduction of vertical COM accelerations, metachronal coordination patterns imply more evenly distributed ground forces, reduce required force peaks and can increase energy efficiency if elastic mechanisms are not applicable (cp. [23]). They also prevent lateral bracing between front and middle legs and the risk of outward slipping, which could cause severe disturbance. Accordingly, such patterns seem to be particularly advantageous on slippery substrates.

Additionally, metachronal leg coordination increases the temporal overlap of the consecutive sets of legs. Therefore permanent ground contact of at least some legs is permitted, although stride lengths increase and duty factors decrease significantly (Figs. 4, 5, and 6). A permanent connection between substrate and some walking legs prevents interruption of proprioceptive information about the animal’s position with respect to the ground and may increase controllability of basically interference-prone locomotion (cp. [71]).

## Conclusion

At high running speeds, when the vertical amplitudes of the body and therefore the use of elasticity are limited, cockroaches avoid the disadvantages of bouncing by temporal dissociation of the alternating sets of legs which might also cause reduced metabolic costs and increased dynamic stability in the horizontal plane. In other words, the change from the alternating tripodal to a metachronal gait pattern at high running speeds can help arthropods to avoid overstraining involved muscles and may facilitate energy efficient high speed locomotion at the same time. Since similar kinematic adaptations were found in a range of unrelated species the high speed metachronal gait is probably widely used by legged terrestrial arthropods and may be a characteristic for fast escape manoeuvres.

### Additional files

Additional file 1: Figure S1. COM kinematics over second legs’ strides for alternating tripodal runs on non-slippery substrate. A stride consists of a contact phase and the subsequent swing phase. The black solid line shows the median course of a value and the grey shaded area the interquartile range. First column: fore-aft direction (X); Second column: lateral direction (Y); Third column: vertical direction (Z). First row: distance in m; Second row: velocity in ms^−1^; Third row: acceleration in ms^−2^. **Figure S2.** COM kinematics over second legs’ strides for metachronal runs on non-slippery substrate. A stride consists of a contact phase and the subsequent swing phase. The black solid line shows the median course of a value and the grey shaded area the interquartile range. First column: fore-aft direction (X); Second column: lateral direction (Y); Third column: vertical direction (Z). First row: distance in m; Second row: velocity in ms^−1^; Third row: acceleration in ms^−2^. **Figure S3.** COM kinematics over second legs’ strides for alternating tripodal runs on slippery substrate. A stride consists of a contact phase and the subsequent swing phase. The black solid line shows the median course of a value and the grey shaded area the interquartile range. First column: fore-aft direction (X); Second column: lateral direction (Y); Third column: vertical direction (Z). First row: distance in m; Second row: velocity in ms^−1^; Third row: acceleration in ms^−2^. **Figure S4.** COM kinematics over second legs’ strides for metachronal runs on slippery substrate. A stride consists of a contact phase and the subsequent swing phase. The black solid line shows the median course of a value and the grey shaded area the interquartile range. First column: fore-aft direction (X); Second column: lateral direction (Y); Third column: vertical direction (Z). First row: distance in m; Second row: velocity in ms^−1^; Third row: acceleration in ms^−2^. **Figure S5.** The courses of pitch, yaw and ∠TA over the second legs’ strides. The black solid line shows the median course of an angle and the grey shaded area the interquartile range. Upper row: Alternating tripodal runs on non-slippery substrate; Second row: Metachronal runs on non-slippery substrate; Third row: Alternating tripodal runs on slippery substrate; Fourth row: Metachronal runs on slippery substrate. **Figure S6.** Stride frequencies, duty factors, swing rates and contact rates plotted against running speed for all walking legs and slippery conditions. First row: Stride frequency (*f*_*T*_); Second row: duty factors (*β*); Third row: swing rates (*t*_*S*_^*−1*^); Fourth row: contact rates (*t*_*C*_^*−1*^). Red circles are measured values for runs with metachronal leg coordination while black crosses depict values from alternating tripodal runs. **Figure S7.** Phase shifts between ipsilateral legs and between the contralateral rear legs during alternating tripodal (white) and metachronal (red) runs on non-slippery (left) and slippery substrate. A, E) Phase values for the touch-downs of the fore legs in the stride period of the rear legs. B, F) Phase values for the touch-downs of the middle legs in the stride period of the rear legs. C, G) Phase values for the touch-downs of the fore legs in the stride period of the middle legs. D, H) Phase values for the touch-downs of the contralateral rear legs. **Figure S8.** Stride durations, swing durations and contact durations plotted against running speed for all walking legs. Stride duration: row one and four; contact duration: row two and five; swing duration: row three and six. The rows one to three refer to non-slippery conditions whereas the rows four to six refer to slippery conditions. Red circles are measured values for runs with metachronal leg coordination while black crosses depict values from alternating tripodal runs. Black (alternating runs) and red (metachronal) lines in the upper three rows were calculated on the basis of the linear least squares regressions for *t*_*S*_^*−1*^ and *t*_*C*_^*−1*^ (see Fig. 4). (PDF 2640 kb)

Additional file 2: Typical running sequence with metachronal leg coordination. The sequence is slowed down to 1/10 of the recording speed. The mean running speed was 0.2 ms-1, the mean phase shift between the ipsilateral legs was 0.77 and the mean duty factor was 0.42. (AVI 1512 kb)

Additional file 3: Typical running sequence with tripodal leg coordination (1/10 recording speed). Beginning on frame 80 the animal increases its mean speed within a fraction of a stride from about 0.1 ms-1 towards a mean running speed of 0.16. Before the change the mean phase shift between ipsilateral legs was 0.9 and the mean duty factor was 0.52. With the higher running speed the mean phase shift between the ipsilateral legs decreased to 0.82 and the mean duty factor to 0.45. (AVI 2343 kb)

## Appendix

**Table 1:** Medians, interquartile ranges and statistical comparisons of running speed, vertical position and amplitude of the COM, lateral amplitude of the COM, amplitudes of pitch and yaw, mean values and amplitudes of the angle between thorax and abdomen and the means of the mass specific external mechanical energy (*M*_*COM*_) as described in the methods section. The values for alternating tripodal (alt) and metachronal (met) runs on slippery (s) and non-slippery (ns) substrates were tested against each other via one-way ANOVA. The sample sizes (n) refer to the numbers of examined strides. Significant differences on the 5% level are indicated by black dots

Parameter	Slipperyness	Pattern	Median (Q25/Q75)	Unit	n	Significance			
						ns	ns	s	s
						alt	met	alt	met
running	ns	alt	0.12 (0.09/0.20)	ms^−1^	62		●		
speed	ns	met	0.21 (0.17/0.24)		74	●		●	●
	s	alt	0.16 (0.13/0.19)		74		●		
	s	met	0.18 (0.13/0.20)		69		●		
vertical	ns	alt	3.66 (3.36/4.11)	mm	62				
position	ns	met	3.74 (3.44/4.04)		74				
	s	alt	3.82 (3.38/4.22)		74				
	s	met	3.65 (3.32/4.03)		69				
vertical	ns	alt	0.94 (0.54/1.31)	mm	62		●		●
peak-to-peak	ns	met	0.63 (0.37/0.80)		74	●		●	
amplitude	s	alt	1.02 (0.85/1.30)		74		●		●
	s	met	0.51 (0.36/1.02)		69	●		●	
lateral	ns	alt	1.55 (1.03/2.56)	mm	62				
peak-to-peak	ns	met	1.73 (1.05/2.21)		74				
amplitude	s	alt	1.55 (1.17/2.23)		74				
	s	met	1.42 (1.04/1.99)		69				
pitch	ns	alt	7.5 (4.6/10.1)	°	62		●		●
peak-to-peak	ns	met	4.5 (3.1/7.0)		74	●			
amplitude	s	alt	5.9 (3.8/8.0)		74				●
	s	met	4.6 (2.6/7.2)		69	●		●	
yaw	ns	alt	8.0 (4.9/10.9)	°	62				
peak-to-peak	ns	met	6.8 (4.6/10.3)		74				
amplitude	s	alt	8.1 (5.5/10.6)		74				
	s	met	8.0 (4.7/10.6)		69				
∠TA	ns	alt	172.0 (168.2/172.8)	°	62		●		●
	ns	met	167.1 (156.3/168.6)		74	●		●	
	s	alt	168.0 (165.8/172.6)		74		●		●
	s	met	163.6 (159.3/165.5)		69	●		●	
∠TA	ns	alt	4.5 (3.1/6.5)	°	62				
peak-to-peak	ns	met	4.4 (2.8/5.8)		74				
amplitude	s	alt	4.7 (3.3/6.5)		74				
	s	met	4.1 (3.1/5.3)		69				
*M*_*COM*_	ns	alt	1.86 (1.29/2.46)	J·kg^−1^·m^−1^	62		●		●
	ns	met	1.47 (1.07/1.81)		74	●		●	
	s	alt	1.82 (1.57/2.34)		74		●		●
	s	met	1.63 (1.13/2.01)		69	●		●	

## Abbreviations

∠TA: Ventral angle between thorax and abdomen
COM: Centre of mass
*f*_*T*_: Stride frequency, i.e. the inverse of the stride duration *t*_*T*_
*M*_*COM*_: Mass specific external mechanical energy per unit distance
*s*_*C*_: Distance travelled by the COM during contact
*s*_*T*_: Distance travelled by the COM during stride
*t*_*C*_^*−1*^: Contact rate, i.e. the inverse of the contact duration *t*_*C*_
*t*_*S*_^*−1*^: Swing rate, i.e. the inverse of the swing duration *t*_*S*_
TSF: Tripod synchrony factor

## References

[1] Bowerman RF (1975). The control of walking in the scorpion. J Comp Physiol.

[2] Ting LH, Blickhan R, Full RJ (1994). Dynamic and static stability in hexapedal runners. J Exp Biol.

[3] Spagna JC, Goldman DI, Lin PC, Koditschek DE, Full RJ (2007). Distributed mechanical feedback in arthropods and robots simplifies control of rapid running on challenging terrain. Bioinspir Biomim.

[4] Blickhan R, Full RJ (1993). Similarity in multilegged locomotion: bouncing like a monopode. J Comp Physiol A.

[5] Sensenig AT, Shultz JW (2006). Mechanical energy oscillations during locomotion in the harvestman *Leiobunum vittatum* (Opiliones). J Arachnol.

[6] Full RJ, Koditschek DE (1999). Templates and anchors: neuromechanical hypotheses of legged locomotion on land. J Exp Biol.

[7] Wilson DM (1966). Insect walking. Annu Rev Entomol.

[8] Graham D (1972). A behavioural analysis of the temporal organisation of walking movements in the 1st Instar and adult stick insect (*Carausius morosus*). J Comp Physiol.

[9] Wendler G (1964). Laufen und Stehen der Stabheuschrecke *Carausius morosus*: Sinnesborstenfelder in den Beingelenken als Glieder von Regelkreisen. Z Vergl Physiol.

[10] Hughes GM (1952). The co-ordination of insect movements. I: the walking movements of insects. J Exp Biol.

[11] Wosnitza A, Bockemühl T, Dübbert M, Scholz H, Büschges A (2013). Inter-leg coordination in the control of walking speed in drosophila. J Exp Biol.

[12] Mendes CS, Bartos I, Akay T, Márka S, Mann RS (2013). Quantification of gait parameters in freely walking wild type and sensory deprived *Drosophila melanogaster*. elife.

[13] Ward TM, Humphreys WF (1981). Locomotion in burrowing and vagrant wolf spiders (Lycosidae). J Exp Biol.

[14] Biancardi CM, Fabrica CG, Polero P, Loss JF, Minetti AE (2011). Biomechanics of octopedal locomotion: kinematic and kinetic analysis of the spider Grammostola Mollicoma. J Exp Biol.

[15] Full RJ, Tu MS (1990). Mechanics of six-legged runners. J Exp Biol.

[16] Weihmann T (2013). Crawling at high speeds: steady level locomotion in the spider *Cupiennius salei* - global kinematics and implications for Centre of Mass Dynamics. PLoS One.

[17] Bartholomew GA, Lighton JRB, Louw GN (1985). Energetics of locomotion and patterns of respiration in tenebrionid beetles from the Namib Desert. J Comp Physiol B.

[18] Alexander RM, Bennet-Clark HC (1977). Storage of elastic strain energy in muscle and other tissues. Nature.

[19] Sensenig AT, Shultz JW (2003). Mechanics of cuticular elastic energy storage in leg joints lacking extensor muscles in arachnids. J Exp Biol.

[20] Dudek DM, Full RJ (2006). Passive mechanical properties of legs from running insects. J Exp Biol.

[21] Bennet-Clark HC (1975). The energetics of the jump of the locust Schistocerca Gregaria. J Exp Biol.

[22] Minetti AE, Ardig OL, Reinach E, Saibene F (1999). The relationship between mechanical work and energy expenditure of locomotion in horses. J Exp Biol.

[23] Ruina A, Bertram JE, Srinivasan M (2005). A collisional model of the energetic cost of support work qualitatively explains leg sequencing in walking and galloping, pseudo-elastic leg behavior in running and the walk-to-run transition. J Theor Biol.

[24] Full RJ, MAR K (1992). Drag and lift on running insects. J Exp Biol.

[25] Full RJ, Tu MS (1991). Mechanics of a rapid running insect: two-, four- and six-legged locomotion. J Exp Biol.

[26] Wahl V, Pfeffer SE, Wittlinger M (2015). Walking and running in the desert ant Cataglyphis fortis. J Comp Physiol A Neuroethol Sens Neural Behav Physiol.

[27] Reinhardt L, Weihmann T, Blickhan R (2009). Dynamics and kinematics of ant locomotion: do wood ants climb on level surfaces?. J Exp Biol.

[28] Weihmann T, Blickhan R. Comparing inclined locomotion in a ground-living and a climbing ant species: sagittal plane kinematics. J Comp Physiol A Neuroethol Sens Neural Behav Physiol. 2009:198:1011–20. 10.1007/s00359-009-0475-y 19756648

[29] Delcomyn F (1971). The locomotion of the cockroach *Periplaneta americana*. J Exp Biol.

[30] Gatesy SM, Biewener A (1991). Bipedal locomotion: effects of speed, size and limb posture in birds and humans. J Zool Lond.

[31] Cavagna GA, Heglund NC, Taylor CR (1977). Mechanical work in terrestrial locomotion: two basic mechanisms for minimizing energy expenditure. Am J Phys.

[32] Heglund NC, Taylor CR (1988). Speed, stride frequency and energy cost per stride: how do they change with body size and gait?. J Exp Biol.

[33] Heglund NC, Taylor CR, McMahon TA (1974). Scaling stride frequency and gait to animal size: mice to horses. Science.

[34] Weihmann T, Goetzke HH, Günther M (2015). Requirements and limits of anatomy-based predictions of locomotion in terrestrial arthropods with emphasis on arachnids. J Paleontol.

[35] Günther M, Weihmann T (2012). Climbing in hexapods: a plain model for heavy slopes. J Theor Biol.

[36] Le Jeune TM, Willems PA, Heglund NC (1998). Mechanics and energetics of human locomotion on sand. J Exp Biol.

[37] Bohn HF, Federle W (2004). Insect aquaplaning: nepenthes pitcher plants capture prey with the peristome, a fully wettable water-lubricated anisotropic surface. PNAS.

[38] Clemente CJ, Federle W (2008). Pushing versus pulling: division of labour between tarsal attachment pads in cockroaches. Proc Biol Sci.

[39] Bender JA, Simpson EM, Tietz BR, Daltorio KA, Quinn RD, Ritzmann RE (2011). Kinematic and behavioral evidence for a distinction between trotting and ambling gaits in the cockroach *Blaberus discoidalis*. J Exp Biol.

[40] Full RJ, Blickhan R, Ting LH (1991). Leg design in hexapedal runners. J Exp Biol.

[41] Kram R, Wong B, Full RJ (1997). Three-dimensional kinematics and limb kinetic energy of running cockroaches. J Exp Biol.

[42] Bullock JMR, Federle W (2011). The effect of surface roughness on claw and adhesive hair performance in the dock beetle Gastrophysa Viridula. Insect Science.

[43] Voigt D, Schuppert JM, Dattinger S, Gorb SN (2008). Sexual dimorphism in the attachment ability of the Colorado potato beetle Leptinotarsa Decemlineata (Coleoptera : Chrysomelidae) to rough substrates. J Insect Physiol.

[44] Dai Z, Gorb SN, Schwarz U (2002). Roughness-dependent friction force of the tarsal claw system in the beetle *Pachnoda marginata* (Coleoptera, Scarabaeidae). J Exp Biol.

[45] Anderson B, Shultz J, Jayne B (1995). Axial kinematics and muscle activity during terrestrial locomotion of the centipede Scolopendra heros. J Exp Biol.

[46] Jamon M, Clarac F (1995). Locomotor patterns in freely moving crayfish (Procambarus Clarkii). J Exp Biol.

[47] Velasco MJ, Philipp B (2009). Circular statistics toolbox for Matlab.

[48] Weihmann T, Karner M, Full RJ, Blickhan R (2010). Jumping kinematics in the wandering spider Cupiennius salei. J Comp Physiol A Neuroethol Sens Neural Behav Physiol.

[49] Spagna JC, Valdivia EA, Mohan V (2011). Gait characteristics of two fast-running spider species (*Hololena adnexa* and *Hololena curta*), including an aerial phase (Araneae: Agelenidae). J Arachnol.

[50] Blickhan R, Full RJ (1987). Locomotion energetics of the ghost crab: II mechanics of the center of mass. J Exp Biol.

[51] Ahn A, Furrow E, Biewener A (2004). Walking and running in the red-legged running frog, *Kassina maculata*. J Exp Biol.

[52] Reinhardt L, Blickhan R (2014). Level locomotion in wood ants: evidence for grounded running. J Exp Biol.

[53] Shultz JW (1987). Walking and surface film locomotion in terrestrial and semiaquatic spiders. J Exp Biol..

[54] Schmitt J, Garcia M, Razo RC, Holmes P, Full RJ (2002). Dynamics and stability of legged locomotion in the horizontal plane: a test case using insects. Biol Cybern.

[55] Cruse H, Dürr V, Schmitz J (2007). Insect walking is based on a decentralized architecture revealing a simple and robust controller. Philos Transact A Math Phys Eng Sci.

[56] Nishii J (2006). An analytical estimation of the energy cost for legged locomotion. J Theor Biol.

[57] Marsh RL, Ellerby DJ, Carr JA, Henry HT, Buchanan CI (2004). Partitioning the Energetics of walking and running: swinging the limbs is expensive. Science.

[58] Fedak MA, Heglund NC, Taylor CR (1982). Energetics and mechanics of terrestrial locomotion. II. Kinetic energy changes of the limbs and body as a function of speed and body size in birds and mammals. J Exp Biol.

[59] Farley CT, Taylor CR (1991). A mechanical trigger for the trot-gallop transition in horses. Science.

[60] Hooper SL, Guschlbauer C, Blumel M, Rosenbaum P, Gruhn M, Akay T, Buschges A (2009). Neural control of unloaded leg posture and of leg swing in stick insect, cockroach, and mouse differs from that in larger animals. J Neurosci.

[61] Ache J, Matheson T (2013). Passive joint forces are tuned to limb use in insects and drive movements without motor activity. Curr Biol.

[62] Reilly SM, McElroy EJ, Biknevicius AR (2007). Posture, gait and the ecological relevance of locomotor costs and energy-saving mechanisms in tetrapods. Zoology.

[63] Armstrong RB, Ogilvie RW, Schwane JA (1983). Eccentric exercise-induced injury to rat skeletal muscle. J Appl Physiol Respir Environ Exerc Physiol.

[64] Lindstedt SL, LaStayo PC, Reich TE (2001). When active muscles lengthen: properties and consequences of eccentric contractions. Physiology.

[65] Keller TS, Weisbrger AM, Ray JL, Hasan SS, Shiavi RG, Spengler DM (1996). Relationship between vertical ground reaction force and speed during walking, slow jogging, and running. Clin Biomech.

[66] McMahon TA, Cheng GC (1990). The mechanics of running: how does stiffness couple with speed?. J Biomech.

[67] Blickhan R (1989). The spring-mass model for running and hopping. J Biomech.

[68] Günther M, Weihmann T (2011). The load distribution among three legs on the wall: model predictions for cockroaches. Arch Appl Mech.

[69] Cavagna GA, Saibene FP, Margaria R (1964). Mechanical work in running. J Appl Physiol.

[70] Schmitt J, Holmes P (2000). Mechanical models for insect locomotion: dynamics and stability in the horizontal plane-II. Application. Biol Cybern.

[71] Sponberg S, Full RJ (2008). Neuromechanical response of musculo-skeletal structures in cockroaches during rapid running on rough terrain. J Exp Biol.

[72] Schaefer PL, Kondagunta GV, Ritzmann RE (1994). Motion analysis of escape movements evoked by tactile stimulation in the cockroach Periplaneta Americana. J Exp Biol.

[73] Carbonell CS (1947). The thoracic muscles of the cockroach *Periplaneta americana* (L.).

[74] Weihmann T. Biomechanische Analyse der ebenen Lokomotion von *Ancylometes bogotensis* (Keyserling, 1877) (Chelicerata, Arachnida, Lycosoidea). doctoral thesis. Friedrich Schiller Universität. Jena: Biologisch-Pharmazeutische Fakultät; 2007.

[75] Manton SM (1972). The evolution of arthropodan locomotiory mechanisms part 10. Locomotory habits, morphology and evolution of the hexapod classes. Zool J Linnean Soc.

[76] Smolka J, Byrne MJ, Scholtz CH, Dacke M (2013). A new galloping gait in an insect. Curr Biol.

[77] Alexander R (1991). Energy-saving mechanisms in walking and running. J Exp Biol.

[78] Li C, Zhang T, Goldman DI (2013). A terradynamics of legged locomotion on granular media. Science.

[79] Dickinson MH, Farley CT, Full RJ, Koehl MAR, Kram R, Lehman S (2000). How animals move: an integrative view. Science.

