# Supplementary material for: Speed dependent phase shifts and gait changes in cockroaches running on substrates of different slipperiness
Source: Front Zool. 2017 Dec 6;14:54. doi: 10.1186/s12983-017-0232-y (PMC5719566; doi:10.1186/s12983-017-0232-y)
Supplement: Supplementary file 1 — COM kinematics over second legs’ strides for alternating tripodal runs on non-slippery substrate. A stride consists of a contact phase and the subsequent swing phase. The black solid line shows the median course of a value and the grey shaded area the interquartile range. First column: fore-aft direction (X); Second column: lateral direction (Y); Third column: vertical direction (Z). First row: distance in m; Second row: velocity in ms−1; Third row: acceleration in ms−2. Figure S2. COM kinematics over second legs’ strides for metachronal runs on non-slippery substrate. A stride consists of a contact phase and the subsequent swing phase. The black solid line shows the median course of a value and the grey shaded area the interquartile range. First column: fore-aft direction (X); Second column: lateral direction (Y); Third column: vertical direction (Z). First row: distance in m; Second row: velocity in ms−1; Third row: acceleration in ms−2. Figure S3. COM kinematics over second legs’ strides for alternating tripodal runs on slippery substrate. A stride consists of a contact phase and the subsequent swing phase. The black solid line shows the median course of a value and the grey shaded area the interquartile range. First column: fore-aft direction (X); Second column: lateral direction (Y); Third column: vertical direction (Z). First row: distance in m; Second row: velocity in ms−1; Third row: acceleration in ms−2. Figure S4. COM kinematics over second legs’ strides for metachronal runs on slippery substrate. A stride consists of a contact phase and the subsequent swing phase. The black solid line shows the median course of a value and the grey shaded area the interquartile range. First column: fore-aft direction (X); Second column: lateral direction (Y); Third column: vertical direction (Z). First row: distance in m; Second row: velocity in ms−1; Third row: acceleration in ms−2. Figure S5. The courses of pitch, yaw and ∠TA over the second legs’ strides. The black sol [file 12983_2017_232_MOESM1_ESM.pdf]

Supplementary material for the article

"Speed dependent phase shifts and gait changes in cockroaches running on substrates of different slipperiness."

Authors: T. Weihmann; E. Pycroft; PG. Brun

Supplementary tables:

Tab. S1 Medians, inter quartile ranges and statistical comparisons of anteroposterior touch down positions (AEP) , take off positions (PEP) , contact lengths (s_C) and lateral distances of the feet with respect to the COM for all walking legs. The values for alternating tripodal (alt) and metachronal (met) runs on slippery (s) and non-slippery (ns) substrates were tested against each other via one-way ANOVA and Tukey-Kramer post-hoc tests. The sample sizes (n) refer to the numbers of examined strides. Significant differences on the 5% level are indicated by black dots.

parameter	slippery-ness	pattern	median (q25/q75)	unit	n	significance			
						ns	ns	s	s
						alt	met	alt	met
AEP	ns	alt	9.0 (7.9/10.3)	mm	78		●		●
fore legs	ns	met	11.4 (10.2/12.8)		79	●		●	
	s	alt	9.4 (8.3/10.2)		57		●		●
	s	met	10.7 (9.2/12.2)		106	●		●	
AEP	ns	alt	3.4 (2.1/4.8)	mm	77				
middle legs	ns	met	4.7 (3.0/5.8)		82			●	
	s	alt	4.2 (2.6/5.2)		58		●		
	s	met	4.3 (2.9/6.1)		103				
AEP	ns	alt	-7.9 (-6.2/-9.4)	mm	77				
hind legs	ns	met	-7.3 (-5.0/-8.6)		84				
	s	alt	-7.6 (-6.1/-9.0)		61				
	s	met	-7.2 (-5.8/-8.6)		104				
PEP	ns	alt	3.3 (2.2/4.9)	mm	78		●		●
fore legs	ns	met	5.2 (4.3/6.9)		79	●		●	
	s	alt	4.0 (2.7/5.3)		57		●		●
	s	met	5.1 (4.0/6.3)		106	●		●	
PEP	ns	alt	-2.4 (-0.8/-3.9)	mm	77				
middle legs	ns	met	-2.1 (-0.2/-3.6)		82				
	s	alt	-1.4 (0.4/-3.0)		58				
	s	met	-1.8 (-0.7/-2.9)		103				
PEP	ns	alt	-13.2 (-11.7/-14.7)	mm	77				
hind legs	ns	met	-12.9 (-11.7/-14.0)		84				
	s	alt	-12.3 (-11.3/-13.4)		61				
	s	met	-12.9 (-11.8/-13.8)		104				
s_C	ns	alt	5.6 (4.9/6.6)	mm	78			●	
fore legs	ns	met	6.0 (5.0/7.0)		79			●	
	s	alt	5.1 (4.4/6.0)		57	●	●		
	s	met	5.9 (4.9/6.7)		106				
s_C	ns	alt	6.0 (4.8/7.3)	mm	77			●	
middle legs	ns	met	6.6 (5.7/7.3)		82			●	
	s	alt	4.8 (4.3/6.0)		58	●	●		●
	s	met	6.0 (4.8/7.3)		103			●	
s_C	ns	alt	5.7 (4.8/6.5)	mm	77			●	
hind legs	ns	met	6.0 (5.0/6.7)		84			●	
	s	alt	4.8 (4.1/5.5)		61	●	●		●
	s	met	5.7 (4.8/6.5)		104			●	
lateral dist.	ns	alt	5.8 (4.6/6.9)	mm	78				
fore legs	ns	met	5.4 (3.7/7.0)		79				
	s	alt	6.2 (4.9/7.2)		57				
	s	met	5.5 (4.2/7.1)		106				
lateral dist.	ns	alt	8.8 (7.6/9.9)	mm	77				
middle legs	ns	met	8.7 (8.0/9.7)		82				
	s	alt	8.4 (7.6/9.6)		58				
	s	met	8.8 (7.6/9.9)		103				
lateral dist.	ns	alt	8.0 (6.4/9.7)	mm	77				
hind legs	ns	met	7.6 (6.8/9.1)		84				
	s	alt	8.4 (6.8/10.1)		61				
	s	met	8.0 (6.4/9.7)		104				

Supplementary figures:

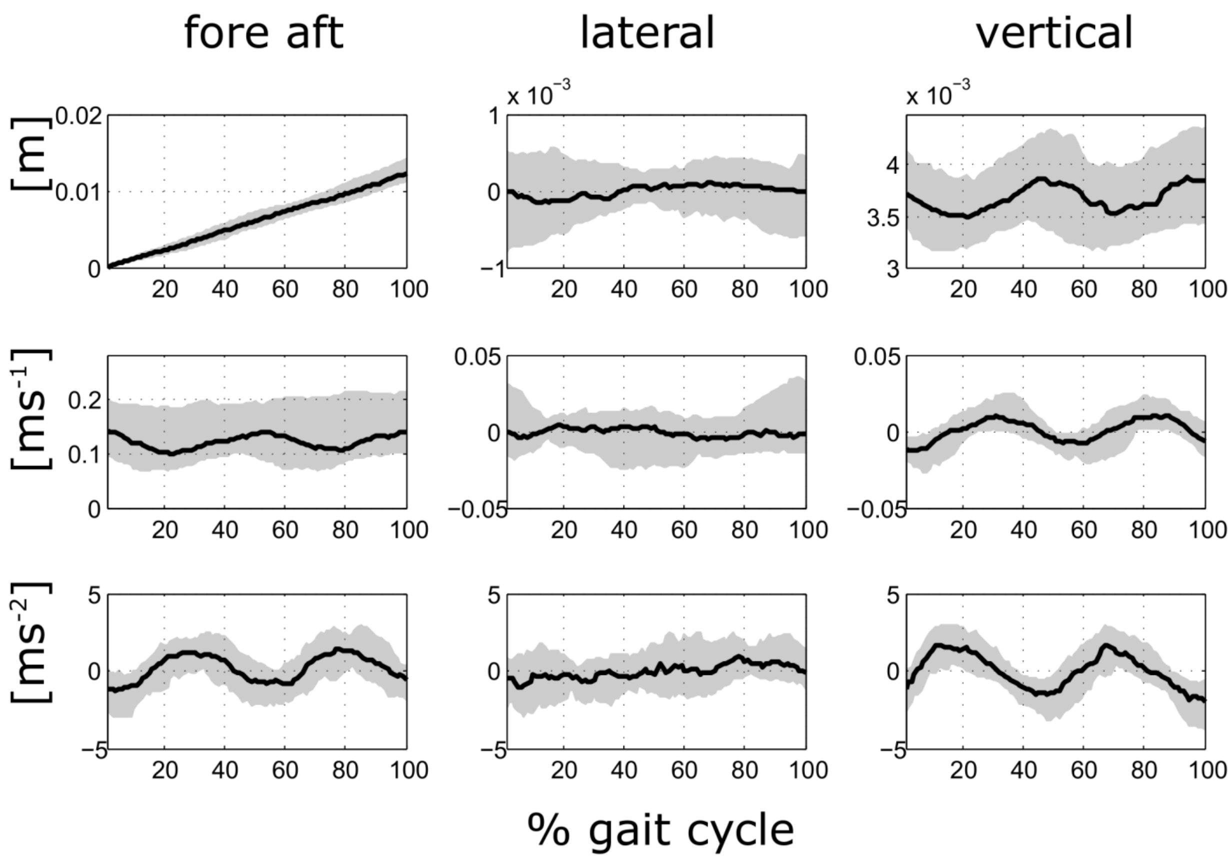

Fig. S1: COM kinematics over second legs' strides for alternating tripodal runs on non-slippery substrate. A stride consists of a contact phase and the subsequent swing phase. The black solid line shows the median course of a value and the grey shaded area the inter quartile range. First column: fore-aft direction (X); Second column: lateral direction (Y); Third column: vertical direction. First row: distance in m; Second row: velocity in ms^{-1}; Third row: acceleration in ms^{-2}.

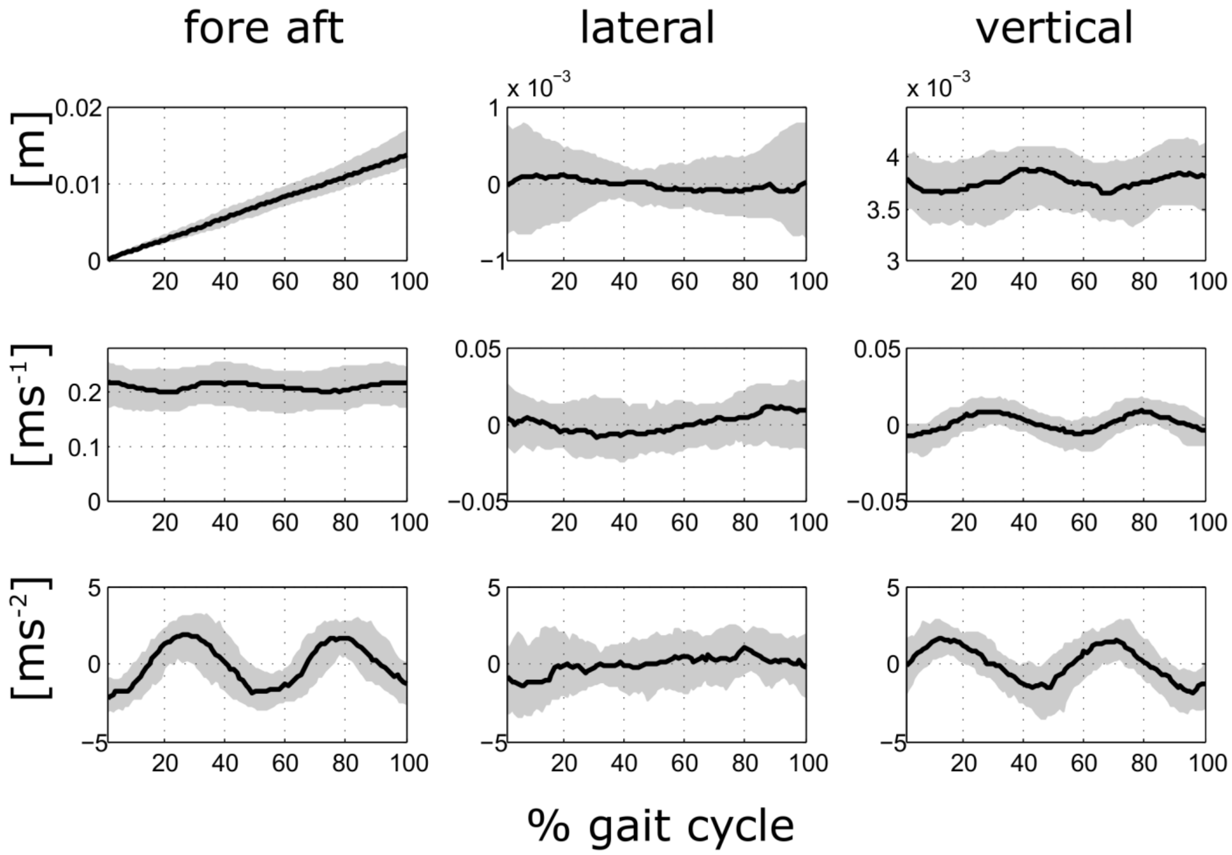

Fig. S2: COM kinematics over second legs' strides for metachronal runs on non-slippery substrate. A stride consists of a contact phase and the subsequent swing phase. The black solid line shows the median course of a value and the grey shaded area the inter quartile range. First column: fore-aft direction (X); Second column: lateral direction (Y); Third column: vertical direction. First row: distance in m; Second row: velocity in ms^{-1}; Third row: acceleration in ms^{-2}.

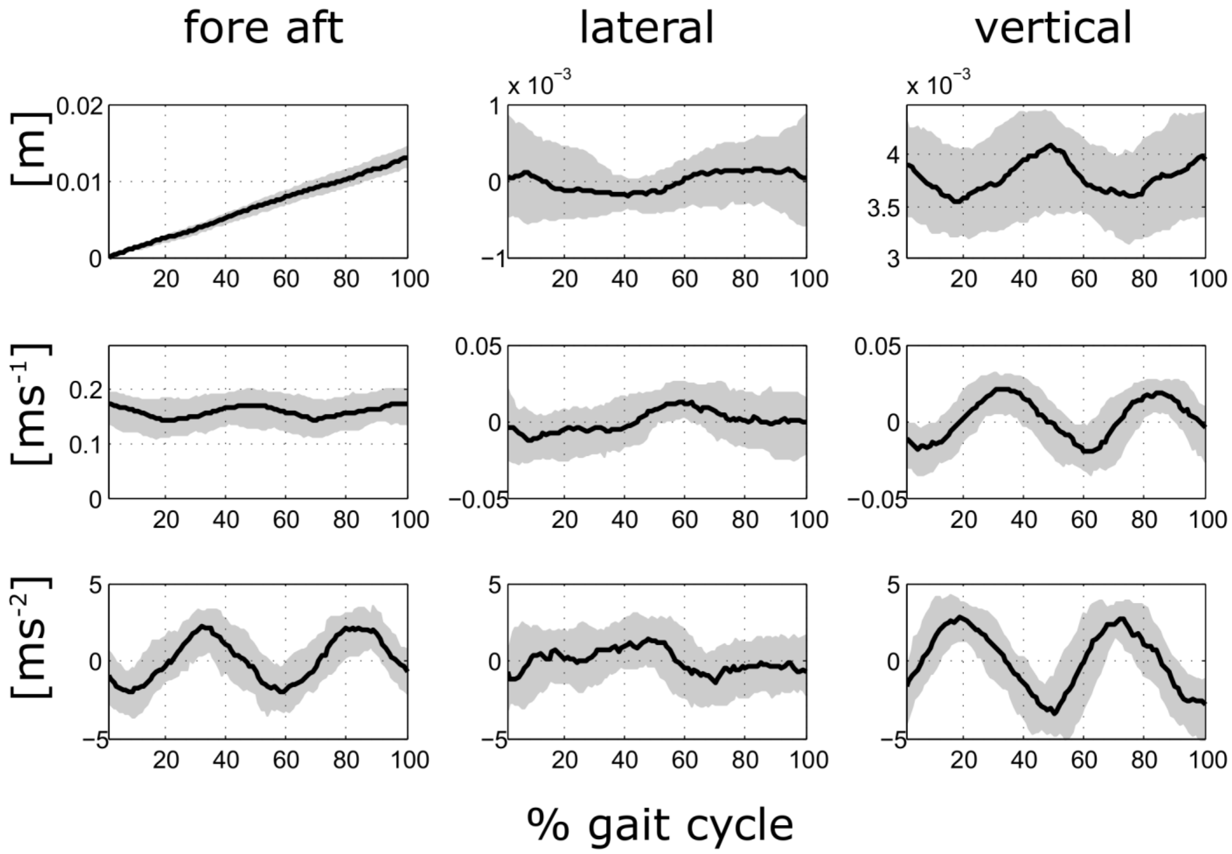

Fig. S3: COM kinematics over second legs' strides for alternating tripodal runs on slippery substrate. A stride consists of a contact phase and the subsequent swing phase. The black solid line shows the median course of a value and the grey shaded area the inter quartile range. First column: fore-aft direction (X); Second column: lateral direction (Y); Third column: vertical direction. First row: distance in m; Second row: velocity in ms^{-1}; Third row: acceleration in ms^{-2}.

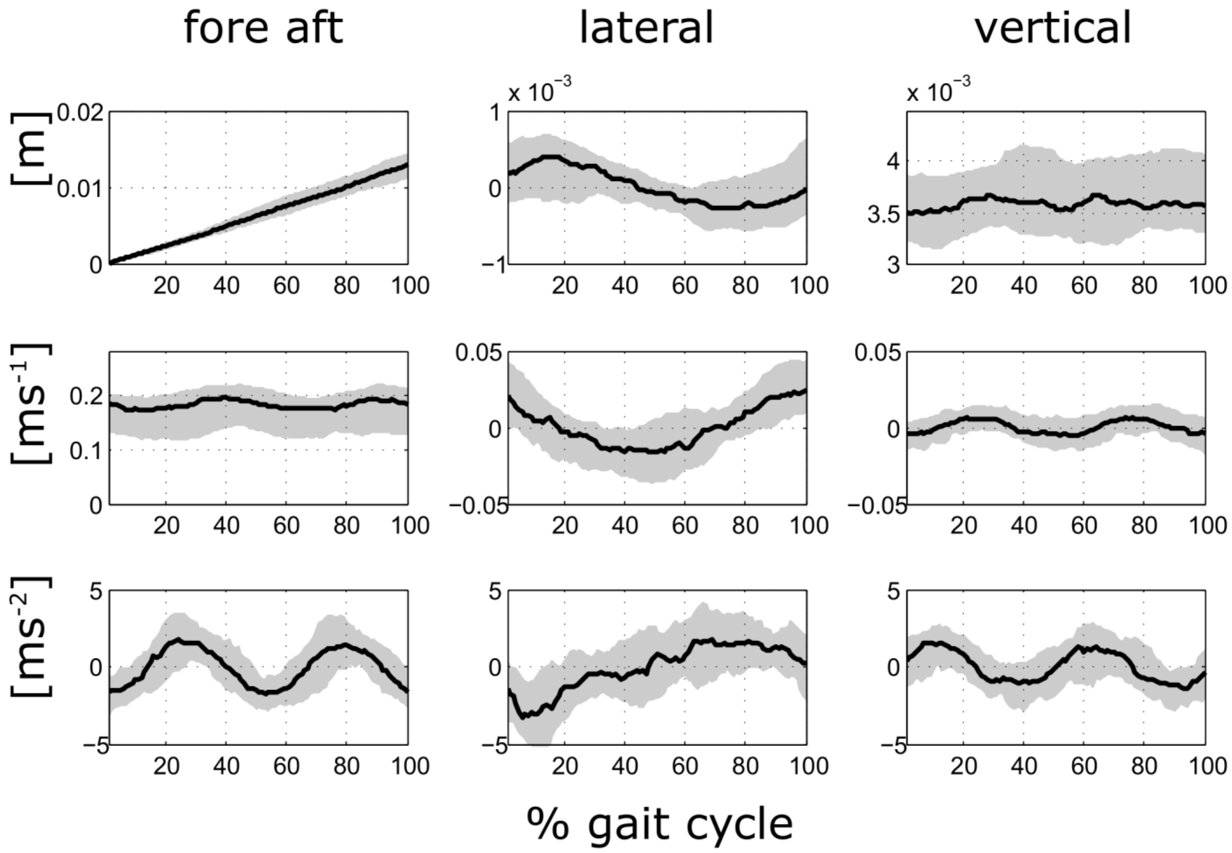

Fig. S4: COM kinematics over second legs' strides for metachronal runs on slippery substrate. A stride consists of a contact phase and the subsequent swing phase. The black solid line shows the median course of a value and the grey shaded area the inter quartile range. First column: fore-aft direction (X); Second column: lateral direction (Y); Third column: vertical direction. First row: distance in m; Second row: velocity in ms^{-1}; Third row: acceleration in ms^{-2}.

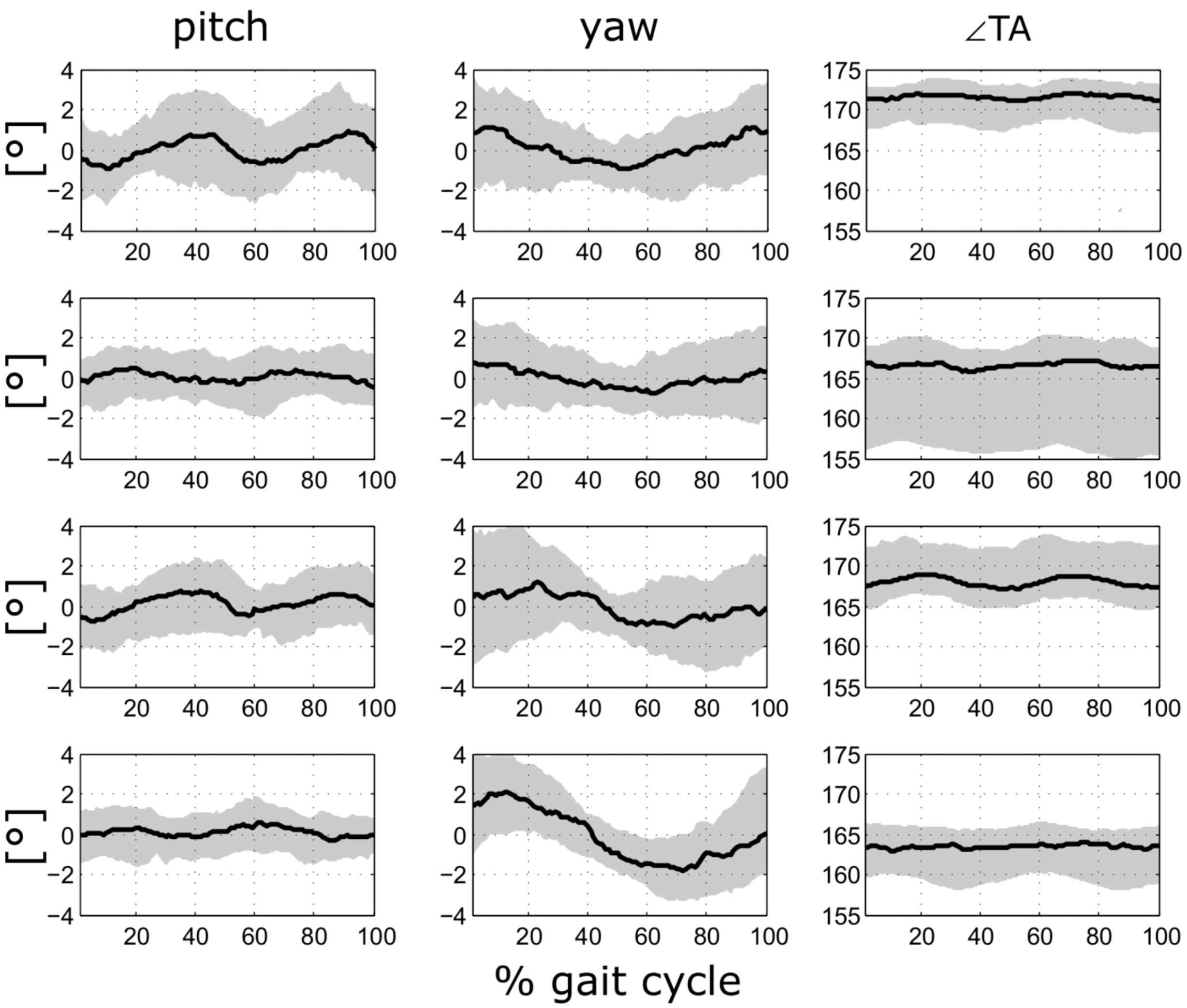

Fig. S5 The courses of pitch, yaw and ∠TA over the second legs' strides. The black solid line shows the median course of an angle and the grey shaded area the inter quartile range. Upper row: Alternating tripodal runs on non-slippery substrate; Second row: Metachronal runs on non-slippery substrate; Third row: Alternating tripodal runs on slippery substrate; Fourth row: Metachronal runs on slippery substrate.

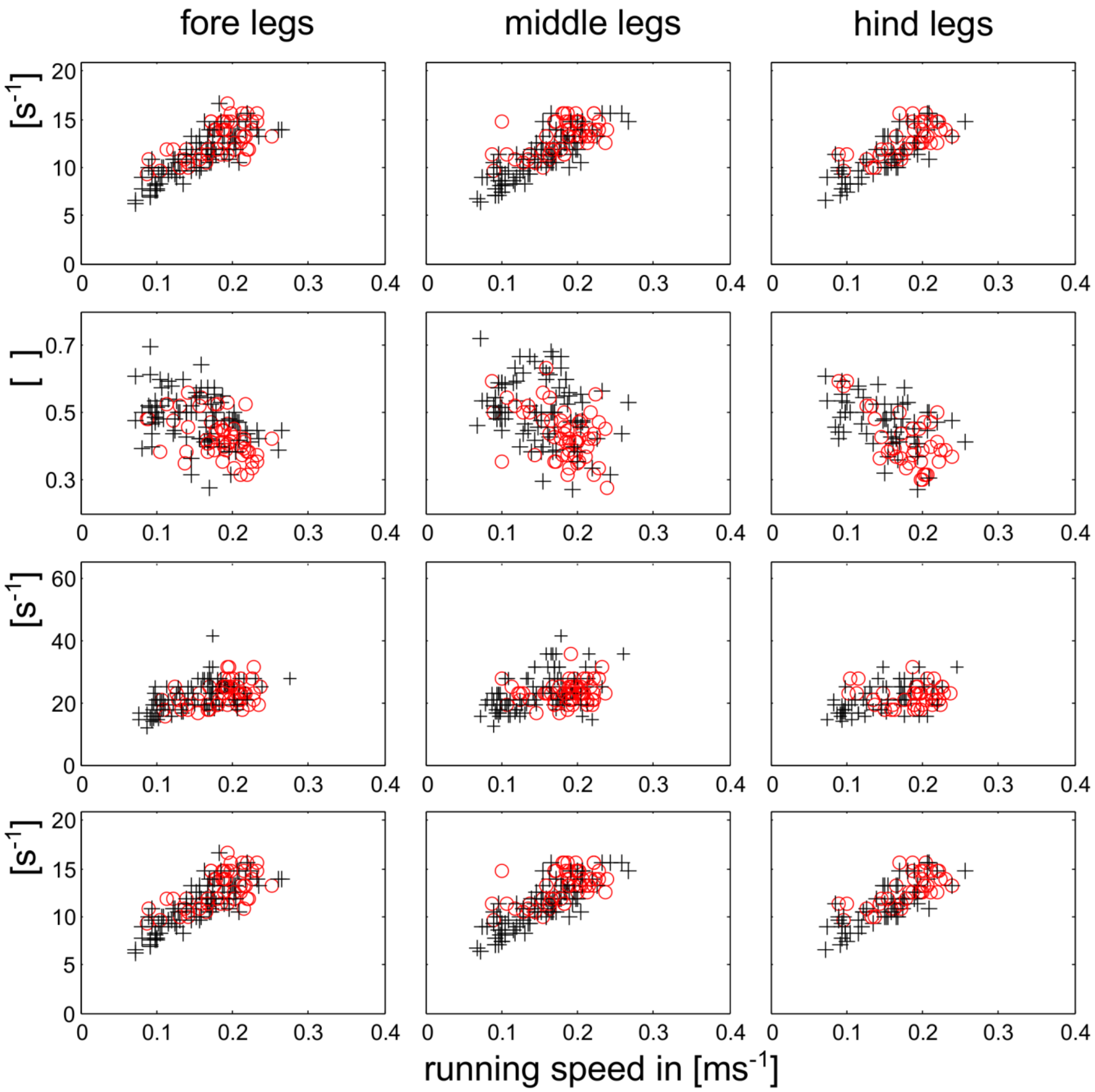

Fig. S6 Stride frequencies, duty factors, swing rates and contact rates plotted against running speed for all walking legs and slippery conditions. First row: Stride frequency (f_T); Second row: duty factors (β); Third row: swing rates (t_S^{-1}); Fourth row: contact rates (t_C^{-1}). Red circles are measured values for runs with metachronal leg coordination while black crosses depict values from alternating tripodal runs.

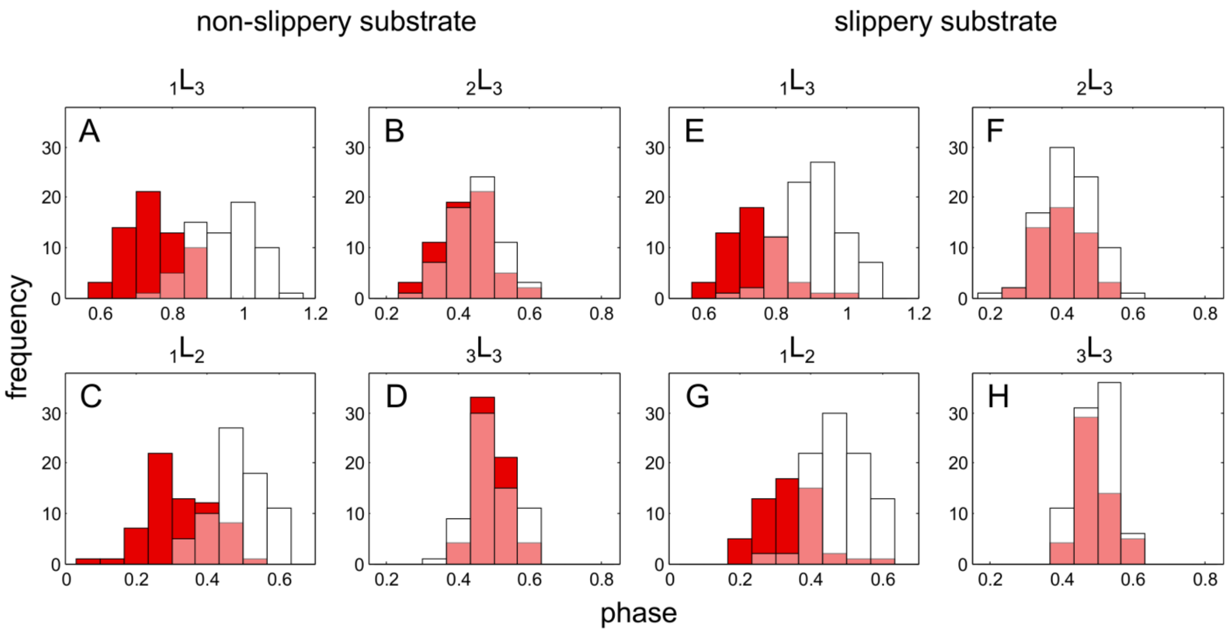

Fig. S7 Phase shifts between ipsilateral legs and between the contralateral rear legs during alternating tripodal (white) and metachronal (red) runs on non-slippery (left) and slippery substrate. A, E) Phase values for the touch-downs of the fore legs in the stride period of the rear legs. B, F) Phase values for the touch-downs of the middle legs in the stride period of the rear legs. C, G) Phase values for the touch-downs of the fore legs in the stride period of the middle legs. D, H) Phase values for the touch-downs of the contralateral rear legs.

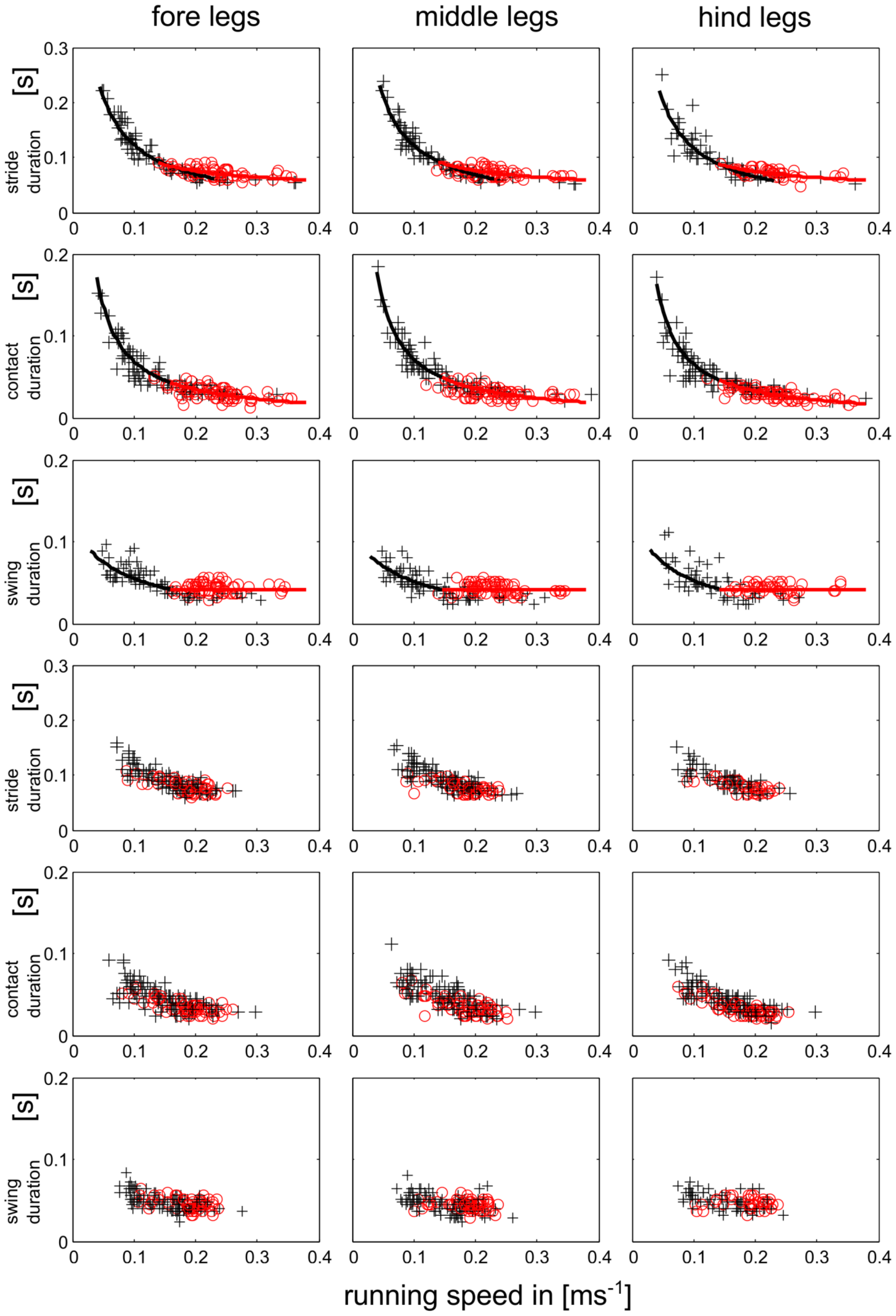

fore legs
middle legs
hind legs
stride duration [s]
contact duration [s]
swing duration [s]
running speed in [ms-1]

Fig. S8 Stride durations, swing durations and contact durations plotted against running speed for all walking legs. Stride duration: row one and four; contact duration: row two and five; swing duration: row three and six. The rows one to three refer to non-slippery conditions whereas the rows four to six refer to slippery conditions. Red circles are measured values for runs with metachronal leg coordination while black crosses depict values from alternating tripodal runs. Black (alternating) and red (metachronal) lines in the upper three rows were calculated on the basis of the linear least squares regressions for t_S^{-1} and t_C^{-1} (see Fig. 5).
